# The proximal enhancer of the *snail* gene mediates negative autoregulatory feedback in *Drosophila melanogaster*

**DOI:** 10.1093/genetics/iyaf058

**Published:** 2025-03-27

**Authors:** Leslie Dunipace, James M McGehee, Jihyun Irizarry, Angelike Stathopoulos

**Affiliations:** Division of Biology and Biological Engineering, California Institute of Technology, 1200 East California Blvd., Pasadena, CA 91125, USA; Division of Biology and Biological Engineering, California Institute of Technology, 1200 East California Blvd., Pasadena, CA 91125, USA; Division of Biology and Biological Engineering, California Institute of Technology, 1200 East California Blvd., Pasadena, CA 91125, USA; Division of Biology and Biological Engineering, California Institute of Technology, 1200 East California Blvd., Pasadena, CA 91125, USA

**Keywords:** *Drosophila melanogaster*, *snail*, *cis*-regulatory mechanisms, shadow enhancers, gene expression levels, autoregulatory feedback, FlyBase

## Abstract

Autoregulatory feedback is a mechanism in which a gene product regulates its own expression, stabilizing gene activity amid noise and environmental changes. In *Drosophila melanogaster*, the gene *snail* encodes a key transcriptional repressor that regulates the expression of many genes during early embryogenesis, including its own expression. This study focuses on Snail occupancy at both distal and proximal enhancers of the *snail* gene to understand the *cis*-regulatory mechanisms involved in autoregulatory control. The coordinated action of these enhancers results in precisely constrained levels of *snail* expression during early embryogenesis. Using genome editing by CRISPR/Cas9, we found that deletion of each enhancer individually is compatible with embryonic viability under normal conditions. However, the double mutant is lethal, suggesting a functional interplay between the 2 enhancers. To gain further insight, we assayed *snail* gene expression levels in fixed embryos. Our results revealed that negative autoregulation of *snail* relies on the proximal enhancer. Moreover, increasing the affinity of binding sites for Dorsal, a transcriptional activator, in the proximal enhancer impaired this autoregulation, suggesting that Snail acts locally to counterbalance Dorsal's input. A mathematical model of *snail* autoregulatory control further supports our findings, reinforcing the view that the proximal enhancer mediates negative autoregulatory feedback, and implicating the distal enhancer in positive autoregulatory feedback. In summary, Snail's role at the proximal enhancer is pivotal for negative autoregulatory control and essential for balancing the activation mediated by the distal enhancer.

## Introduction

Precise control of key transcription factor expression levels is critical for ensuring correct downstream gene expression and proper organism development. Since many developmentally important transcription factors target multiple genes, regulating their levels is essential. One mechanism for modulating gene expression levels is autoregulatory feedback, employed from bacteriophage to humans (Alon 2007). Autoregulatory mechanisms can be positive or negative, direct or indirect, and can involve a single or multiple interacting *cis*-regulatory modules (CRMs) (Crews and Pearson 2009).

Since the same transcription factor can be positively or negatively autoregulated depending on the cell type, most autoregulatory CRMs are unlikely to consist solely of multiple binding sites for the autoregulatory transcription factor. Instead, they are often more complex, involving binding sites for co-regulatory proteins present in specific cell types, or even input from multiple CRMs. The nature of the module(s), strength of DNA-binding site affinity, the number of binding sites, and interactions with other transcription factors all contribute to autoregulatory control (Crews and Pearson 2009; Dunipace et al. 2013).

*Cis*-regulatory control of autoregulating transcription factors has been previously explored in the *Drosophila melanogaster* model system. The *Distal-less* (*Dll*) gene features 1 autoregulatory fragment, LT, which maintains expression in the head and thoracic primordia of the embryo, and another fragment, M, required in the leg disc during the larval stage (Galindo et al. 2011). While LT is sufficient for embryonic expression, both LT and M contribute to maintaining expression in the larva. Therefore, autoregulation of *Dll* requires coordination between multiple enhancers. In contrast, the *Deformed* (*Dfd*) homeobox gene contains several autoregulatory CRMs that each maintain expression in specific subdomains of the *Dfd* ectoderm expression pattern and apparently act individually to effect autoregulation (McGinnis et al. 1990; Lou, Bergson, and McGinnis 1995). However, most of these studies have been done using exogenous reporters which may not properly capture the dynamics of a system where the gene product regulates itself. Advances in genome editing have enabled the creation of specific mutations in enhancers at the endogenous locus, making it easier to analyze how genes accomplish autoregulation at the *cis*-regulatory level (Crews 2017). A recent study using gene editing deleted the autoregulatory enhancer for the gene *fushi tarazu* (*ftz*) at the endogenous locus, finding that this mutation caused premature loss of *ftz* but had little impact on Ftz target gene expression or viability under normal laboratory conditions (Fischer, Graham, and Pick 2024). While these investigations have characterized properties of autoregulatory control, there is no clear consensus on whether each enhancer must mediate its own autoregulatory control, how negative vs positive autoregulation is similar or different, or whether autoregulatory control supports viability.

In *Drosophila melanogaster*, a strong candidate for studying autoregulatory control at the gene locus is *snail* (*sna*). *sna* is an important developmental regulator, exhibits documented negative autoregulation, and also has a *cis*-regulatory system that has been analyzed using exogenous transgenes. *sna* encodes a Zn-finger transcription factor that functions to support epithelial-to-mesenchymal transitions (EMTs) and, along with another transcription factor, Twist (Twi), is necessary for proper gastrulation (Simpson 1983; Nüsslein-Volhard, Wieschaus, and Kluding 1984; Leptin and Grunewald 1990; Lamouille, Xu, and Derynck 2014). Although first discovered in *Drosophila*, this EMT function is conserved in higher animals (Carver et al. 2001). Furthermore, in both *Drosophila* and mice, elevated levels of *sna* have been shown to increase the number of metastases (Tran et al. 2014; Sharpe et al. 2023). Therefore, understandably, multiple mechanisms act to control Sna protein levels, ranging from regulation of transcript levels to protein stability (Muqbil et al. 2014).

Here, we focused on understanding how 2 CRMs function in early embryos to regulate *sna* transcript levels, in particular, through autoregulatory feedback. Previous studies on the *cis*-regulatory control of *sna* utilized small and large exogenous reporter constructs (Ip et al. 1992; Perry et al. 2010; Dunipace, Ozdemir, and Stathopoulos 2011; Ozdemir et al. 2014). From this previous work, 2 enhancers—the proximal (primary) enhancer and the distal (shadow) enhancer—were identified as critical for supporting *sna* expression in the early embryo (Perry et al. 2010; Dunipace, Ozdemir, and Stathopoulos 2011). The expression supported by the 2 enhancers is largely overlapping in both space and time. When both enhancers were simultaneously deleted from a large reporter construct, no *sna* reporter expression was observed in early, pregastrulation embryos (Dunipace, Ozdemir, and Stathopoulos 2011). Live imaging and subsequent quantification demonstrated that expression supported by the 2 enhancers is nonadditive, indicating a cooperative activity between the 2 enhancers (Bothma et al. 2015; Irizarry and Stathopoulos 2021). Further, the levels of *sna* in the presumptive mesoderm are refractory to changes in gene copy, supporting a role for autoregulatory feedback in maintaining transcriptional levels (Boettiger and Levine 2013). Autoregulation is thought to help establish developmental cell fates through bistability, a network motif associated with fast accumulation of transcripts with a sudden locking into steady state (Zhao et al. 2023). Such regulatory control may support the fast, though controlled, production of *sna* with uniform levels necessary to trigger proper gastrulation movements (Lagha et al. 2013). Given the importance of *sna* in development as a transcriptional repressor and its documented dosage compensation, we used genome editing to create targeted mutations in order to gain greater understanding of embryonic *sna* enhancers in their native context and to determine whether negative autoregulatory feedback acts at one or both of these enhancers.

## Materials and methods

### Fly stocks/husbandry and crosses

All flies were reared at 23°C. Embryo collections were done at 25°C. *yw* is used for wild type. All fly stocks used in this study are listed in Supplementary Table 2 in Supplementary File 2. Homozygous mutant lines (*sna1*; *Df; Δdist2.0*; *Δprox1.3_Δdist2.0*) were rebalanced over *CyO,Hb-LacZ* (BDSC#7750) for imaging or *CyO, Act-GFP* (BDSC#36320) for viability studies.

### CRISPR/Cas9-mediated genome modification

For CRISPR/Cas9 deletions within the genome, gRNA constructs were created by modifying the pCFD4 or pCFD5 plasmid (Addgene Plasmid #49411, Port et al. 2014; #73914, Port and Bullock 2016) to target PAM sequences flanking the region to be deleted. Homology-directed repair plasmids were made using pHD-DsRed vector (Addgene plasmid #51434, Gratz et al. 2014), inserting ∼1 kb homology arms on either side of the loxP-flanked DsRed marker. Deletions were made both with and without homology-directed repair. All primers used for cloning are listed in Supplementary Table 3 in Supplementary File 2. A more detailed description is provided in Supplementary Methods in Supplementary File 2 along with sequences of the mutants generated for this study.

### Hybridization chain reaction fluorescent in situ and imaging

Embryos were collected for 2 h at 25°C and allowed to age at the same temperature for 2 more hours, enriching for stage 5 embryos. Hybridization chain reaction (HCR) was performed according to the Molecular Instruments’ HCR RNA-FISH protocol for whole-mount fruit fly embryos, omitting the proteinase K step, and mounted in Invitrogen Slow Fade Gold mounting media (Choi et al. 2018). HCR probes were designed by Molecular Technologies against the whole mRNA transcript to NM_057384.4 (*sna*) and NM_079616.4 (*sim*). A *wt* control was stained alongside all staining sets for comparison between sets stained on different days.

Fluorescent images were acquired using a Zeiss LSM 800 microscope and 20× objective. All HCR samples were collected, stained, and imaged concurrently using the same settings for acquisition when possible. Image processing was done in Zen software. All embryo images shown are max-intensity Z projections of 20 z-stacks (1.8 μM each). Rainbow color maps of embryos were made using ImageJ by importing the max-intensity Z-projection from Zen and applying a custom LUT.

To quantify *sna* levels in the various enhancer mutations, the average fluorescence intensity was quantified in mid-to-late nc 14 embryos. The intensities were then divided by the mean intensity for the control, *yw*, to give the fold change. See Supplementary Methods in Supplementary File 2 for a more detailed description.

### Cuticle preps

Cuticle preps were performed according to standard procedures using lactic acid. Images were collected on either a Zeiss Axioplan microscope with 10× objective for phase contrast images or a Zeiss Imager.Z2 with 20× objective. Minor defects were classified as changes in bristle organization in denticle bands, and major defects were defined as the curled phenotype consistent with *sna* mutants (Nüsslein-Volhard, Wieschaus, and Kluding 1984) or as the loss of denticle bands.

### Viability assays

Embryonic viability was assayed as in Rockwell, Beaver, and Hongay (2019). A more detailed description is provided in Supplementary Methods in Supplementary File 2.

### Modeling

The models were generated using mass action kinetics and a Hill function to write a set of ordinary differential equations (ODEs; Equations 1 and 2). In addition, a model where the Hill coefficient *n* was assumed to go to infinity was generated (Equations 3–5). These equations were analyzed by analytically solving for steady state. The analysis when *n* goes to infinity is included in the main text and Fig. 4. See Supplementary Methods in Supplementary File 2 for a full description of the analytical analysis of Equations 1 and 2 as well as the comparison of a Hill function when *n* equals 4 was compared with when *n* goes to infinity.

### Statistical analysis

Bootstrapping was used for the statistical analysis of the viability assay. First, we took the number of hatched embryos and the total number of embryos, and converted them into binary or true (one) for embryos that hatched (viable) and false (zero) for embryos that did not hatch. Second, we pooled the data for the replicates of each condition. To perform the bootstrap analysis, we calculated the combined mean between the control and a mutant condition. The respective means are then subtracted from the control and mutant condition data, and the combined mean is added to both. This allows the bootstrapping to test the hypothesis that the control and mutant conditions have the same mean by shifting the data to have the same mean. Bootstrapping is performed by randomly drawing with replacement from the shifted control and mutant dataset. The difference between the means of the original control and mutant data is then compared with the difference between the means of the control and mutant bootstrap samples. This was repeated 10 million times. The number of times the absolute value of the difference in means of the bootstrap samples was greater than the absolute value of the difference in means of the original sample divided by the number of bootstrap samples gives the *P*-value. This was repeated for all mutant conditions. Since we made repeated comparisons with the control, we adjusted our *α*-value using the Bonferroni correction. For 95% confidence, the *α*-value is 0.05, and we made 9 comparisons, so the corrected *α*-value is *α*/number of comparisons, or 0.05/9 = 5.56 × 10^−3^. This test compares whether the means are different and is an appropriate test since the mean of binary data is equal to the percent viable for this data.

For the comparisons of the mean levels of *sna* signal intensity, ANOVA was performed followed by Tukey's honestly significant difference (HSD) for multiple comparisons.

## Results and discussion

### Generation of *sna* enhancer deletions using genome engineering

**Fig. 1.**
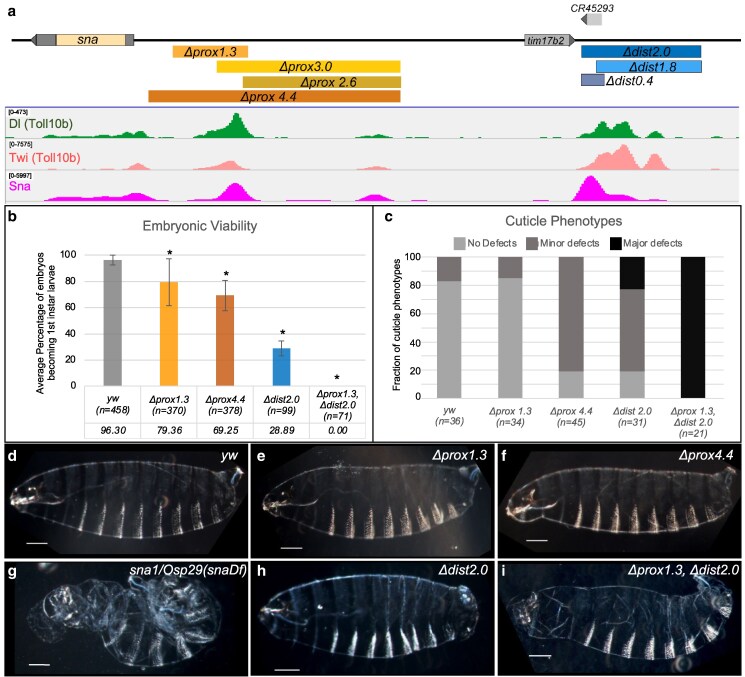
CRISPR/Cas9 mutants removing either the proximal or distal enhancer regions show reduced viability. a) *sna* locus and deletions mapped above ChIP-seq data from previously published datasets [Sna: GSM1689688; toll10b_twi_1: GSM1689698; toll10b_dl_1: GSM1689690 (Koenecke et al. 2016)]. b) Viability of mutants at 25°C shown as the percent of survivors (black lines indicate ±SD). The asterisk represents *P*-values <5.56 × 10^−3^ as computed through bootstrapping (see Materials and methods). c) Frequency of cuticle phenotypes. Minor defects are variations in denticle band bristles, while major phenotypes are lost denticle bands or tail up curvature of the embryos. Total number of embryos counted (*n*) is shown under the genotype label in both b and c. Examples of each classification are shown in Supplementary Fig. 1 in Supplementary File S1. d–i) Example images of cuticles for mutants representing the most common phenotypes observed for each genotype.

To determine where autoregulation is controlled in *sna's* enhancers, mutations and truncations of the proximal (primary) and distal (shadow) enhancers (Perry et al. 2010; Dunipace, Ozdemir, and Stathopoulos 2011) were generated using CRISPR/Cas9 technology (see Materials and methods). The “primary” enhancer (Perry et al. 2010) was defined as the minimal portion that drives expression in a broad, strong pattern in the ventral region of the embryo through a reporter assay (Ip et al. 1992; deleted in *Δprox1.3*). The “proximal” enhancer was defined as an ∼3.8 kb region, delimited by Twi binding in the early embryo, which completely overlaps the primary enhancer sequence (Dunipace, Ozdemir, and Stathopoulos 2011; approximated by *Δprox4.4*). To further dissect this region, 2 other proximal deletions were made: a 2.6-kb deletion upstream of the primary enhancer (*Δprox2.6*), and a 3.0-kb deletion overlapping the *Δprox2.6* completely and extending into the primary enhancer region (*Δprox3.0*) (Fig. 1a). The *Δprox3.0* removes 2 regions where transcription factor binding was detected by chromatin immunoprecipitation coupled with high throughput sequencing (ChIP-seq). These data indicate overlapping regions of binding of the activators Dorsal (Dl) and Twi, which are important drivers of *sna* expression, as well as Sna binding (Fig. 1a; Koenecke et al. 2016). For the distal enhancer, 0.4 kb was deleted to remove the peak of Sna binding in ChIP-seq data (*Δdist0.4*), 1.8 kb was deleted to remove the activator portion while leaving the Sna binding (*Δdist1.8*), or the entire enhancer was deleted (*Δdist2.0*; Fig. 1a; Koenecke et al. 2016). In addition, a double deletion was generated that removed both the *Δprox1.3* and *Δdist2.0* in the same allele (*Δprox1.3_Δdist2.0*).

All of these mutations are viable and are maintained as homozygous stocks, except for the *Δdist2.0* and the *Δprox1.3_Δdist2.0.* The double mutant *Δprox1.3_Δdist2.0* is zygotic lethal, while the *Δdist2.0* is semi-lethal and sterile. From a heterozygous cross only 3–5% of the *Δdist2.0*, adults were homozygous, compared with an expected 33%, accounting for the fact that *CyO*/*CyO* flies are not viable (*n* = 1,249). The loss of viability in the *Δdist2.0* and the *Δprox1.3_Δdist2.0* mutant lines likely not only relates to effects on *sna* expression in the early embryo but also could stem from defects that manifest later in development.

### Both the proximal and distal enhancers contribute to viability

Sna is a key regulator of mesoderm specification and invagination in early embryonic development, and *sna* mutants show severe defects in cuticle formation and do not survive beyond embryogenesis (Grau, Carteret, and Simpson 1984; Nüsslein-Volhard, Wieschaus, and Kluding 1984). Hatched larvae from the CRISPR/Cas9 mutants were counted to test the effects of the enhancer deletions on embryonic viability, as even viable mutations could be associated with decreased survivorship (see Materials and methods). Proximal enhancer deletions had moderate decreases in viability (∼10–30%), while distal enhancer deletions had more severe decreases in viability (∼15–70%; Fig. 1b, Supplementary Fig. 1a in Supplementary File 1). To further characterize these deletions in terms of patterning phenotypes, cuticle preps were performed and categorized by severity (Fig. 1c–i, Supplementary Fig. 1b–d in Supplementary File 1; see Materials and methods). The *Δdist2.0* and *Δprox1.3_Δdist2.0* had the most severe cuticle phenotypes, while the *Δprox4.4* showed minor defects (Fig. 1c; see also images in Fig. 1d–i and Supplementary Fig. 1c and d in Supplementary File 1). These data show that the combined activity of the proximal and distal enhancers is necessary for proper development of the embryo, as the phenotype of the double mutant (*Δprox1.3_Δdist2.0*) is more severe than deletion of either enhancer individually.

### The *sna* distal enhancer is the predominant driver of *sna* expression in the early embryo

**Fig. 2.**
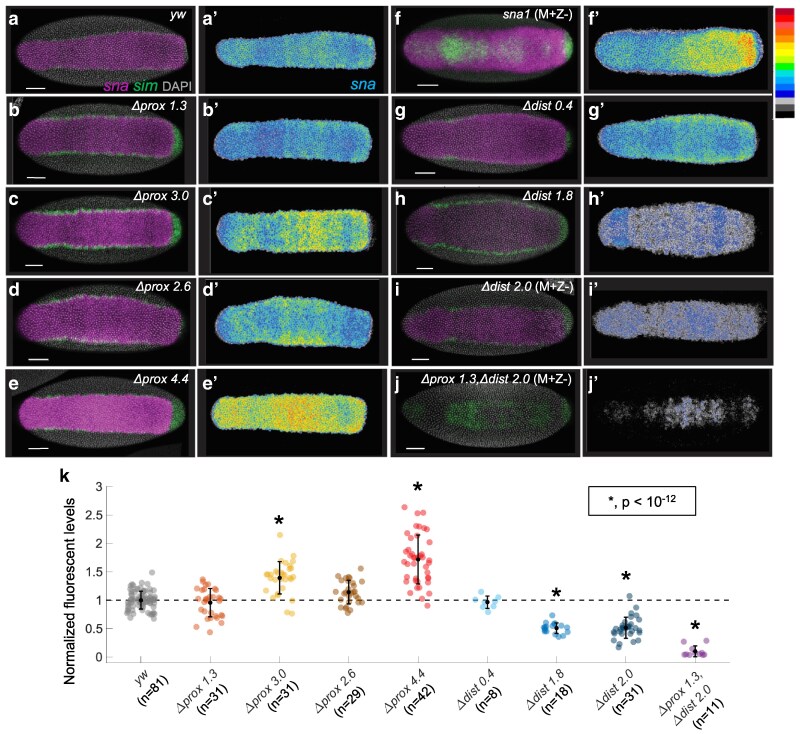
Deletion of the proximal element results in increased expression of *sna*. a–j) Representative images of *sna* (magenta), *sim* (green), and DAPI (gray) for mutant embryos. All embryos in this and subsequent panels are oriented ventral side up, anterior to the left, in mid-to-late nuclear cycle 14. White scale bar indicates 50 µm. All embryos are maternal and zygotic mutants except those that are not homozygous viable and were assayed as maternal + zygotic−, noted in the figure as (M + Z−). a′–j′) *sna* expression displayed using a rainbow color map, where high levels correspond to red, and low levels are black. k) Quantification of *sna* levels in proximal and distal enhancer mutants. Fluorescence levels were quantified by taking the mean intensity in the detected *sna* domain. The mean intensities were then averaged across embryos of the same condition. These values were then divided by the average across embryos of the control, *yw*. Statistical significance was tested using Tukey's HSD for multiple comparisons after performing 1-way ANOVA. One asterisk represents *P*-values <10^−12^ when comparing mutant conditions to the control, *yw*. Total number of embryos measured (*n*) is shown under the genotype label, mean ± SD shown in black.

To test whether transcription levels are altered by these enhancer deletions, *sna* expression was assayed during mid-to-late nuclear cycle (nc) 14, when it is required for proper specification of mesodermal cells. This is the first stage that autoregulatory feedback is thought to function and is critical for normalizing the levels of *sna* expression (Boettiger and Levine 2013). Embryos were fixed and stained using HCR fluorescent in situ hybridization (HCR-FISH; Choi et al. 2018) with probes for both *sna* and *single-minded* (*sim*), a gene repressed by Sna in the ventral mesoderm (Fig. 2a–j; Park and Hong 2012).

**Fig. 3.**
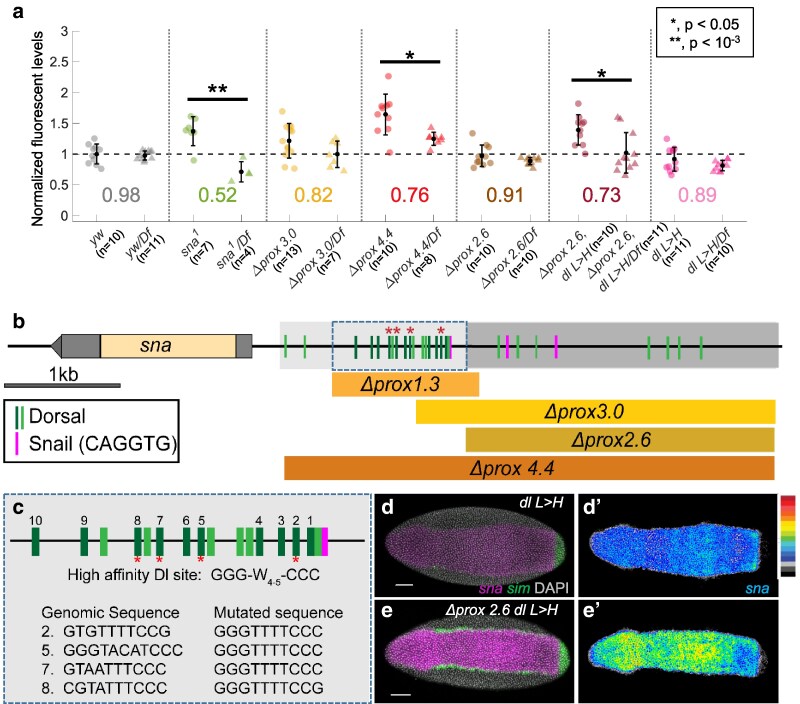
Autoregulatory compensation of *sna* levels occurs when 1 copy is removed, but is reduced when the proximal element is deleted or Dl-binding affinity is altered in a compromised background. a) Quantification of *sna* levels when either 2 copies or 1 copy is present. Quantification was performed as in Fig. 2. Numbers displayed below paired conditions represent the levels of 1 copy (e.g. *yw*/*Df*) divided by the levels of 2 copies (e.g. *yw*/*yw*, which by convention is noted as *yw*). One asterisk represents *P*-values <0.05 and 2 asterisks represent *P*-values <10^−3^ when comparing 1 to 2 copies using Tukey's HSD for multiple comparisons after performing 1-way ANOVA. Total number of embryos measured (*n*) is shown under the genotype label, mean ± SD shown in black. Maternal genotype for all 1 copy crosses is *Df(2L)Osp29*/*CyO.* b) Diagram of the proximal region of the *sna* locus with the deletions mapped below and binding sites for Dl and Sna marked on the chromosome line. Dl-binding sites described in Ip et al. (1992) are marked in dark green, additional Dl-binding sites identified by Jaspar (Rauluseviciute et al. 2024) are shown in light green, and the high-affinity Sna-binding site (CAGGTG; Mauhin et al. 1993) is marked with pink. The region removed by the *Δprox2.6* is marked with a shaded dark gray box and changes in Dl-binding sites (*dl L > H*) indicated with red asterisks. c) Magnified view of the region inside the hatched box in (b) that is retained in *Δprox2.6*. The Dl-binding sites are labeled 1–10 in accordance with Ip et al. (1992). The Dl sites that were mutated are denoted with a red star and the genomic sequence for each is shown next to the high-affinity site that they were mutated into. d, e) Representative images of *sna* (magenta), *sim* (green), and DAPI (gray) in *dl L > H* (b) or *Δprox2.6_dl L > H* (c). d′, e′) The same images as in (b) and (c) except with *sna* expression shown on a rainbow color map with high levels in red and low levels in black.

Although largely stereotyped, the width of the *sna* expression domain is known to scale to the size of the dorsal–ventral axis of the embryo, with larger embryos exhibiting slightly wider *sna* expression patterns. The natural variation in the width of the *sna* expressing cells during nc 14 is from 16 to 20 nuclei, or ∼112 to 140 µm (Garcia et al. 2013; Supplementary Fig. 2 in Supplementary File 1, *yw*). This population-level variability is captured in the range of widths exhibited by the *yw* embryos and the majority of the CRISPR/Cas9 mutant lines, with only 3 of the lines showing a significant difference in width (Supplementary Fig. 2 in Supplementary File 1). The increases in width associated with the *dl L > H* (see section below and Fig. 3c) and *Δdist1.8* mutants could relate to increased Dl-binding site affinity (Jiang and Levine 1993) and loss of Suppressor of Hairless repressor input (Ozdemir et al. 2014), respectively; both leading to increased Dl-dependent activation. In turn, the decrease in width associated with mutant *Δprox3.0* could relate to decreased Dl-dependent activation, since some Dl-binding sites are removed (e.g. see Fig. 1a). However, since *Δprox4.4* encompasses the *prox3.0* region but does not show the same effect on the width as *Δprox3.0*, there must be other important factors at play controlling *sna* width.

To focus specifically on the levels of *sna* expression, we measured the average intensity of fluorescence expression detected by in situ, which normalizes for the slight changes in expression domain exhibited both within and between genotypes. Compared with control (i.e. *yw*), the *Δprox3.0* and *Δprox4.4* had increased levels of *sna* (Fig. 2k, *P* < 10^−12^), while the *Δdist1.8* and *Δdist2.0* had reduced levels of *sna* (Fig. 2k, *P* < 10^−12^). The *Δprox1.3_Δdist2.0* supported only patchy, low-level expression (Fig. 2j, j′, and k, *P* < 10^−12^). The ability of Sna to repress ectodermal and mesectodermal genes, such as *sim*, in the presumptive mesoderm can be used to further assess the severity of the mutant phenotypes (Hemavathy, Meng, and Ip 1997). All of the mutants had sufficient Sna to repress *sim* in the ventral midline, except for *sna*^*1*^ and the mutant deleting both enhancers, *Δprox1.3_Δdist2.0* (Fig. 2a–j). Thus, functionally, the 2 enhancers appear to be somewhat redundant, but the reduction in *sna* expression associated with the distal deletions suggests that the distal enhancer is the predominant driver of *sna* expression at this stage, which is congruent with previous studies (Dunipace, Ozdemir, and Stathopoulos 2011; Bothma et al. 2015).

### The proximal enhancer contains redundant regions of repression

One possible explanation for why the levels of *sna* are increased in the proximal deletions is that deleting 1.3 to 4.4 kb brings the distal enhancer closer to the *sna* promoter and allows increased activation. Distance-dependent expression has been observed in reporter constructs using the *sna* distal enhancer and a synthetic promoter (Yokoshi, Segawa, and Fukaya 2020). However, for the deletions we generated, deletion size does not correlate with the increase in expression levels. Specifically, the *Δprox1.3* and *Δprox2.6* deletions do not have significant increases in *sna* expression, while the *Δprox3.0*, which is only 400 bp bigger than the *Δprox2.6*, does (Fig. 2k, see also Fig. 1a). Thus, moving the distal closer to the promoter, on its own, may not be sufficient to explain the observed differences.

An alternative explanation is that the proximal enhancer deletions removed sites for repression, resulting in higher levels of *sna* expression. In this interpretation, the *Δprox1.3* and *Δprox2.6* do not exhibit a significant change in expression, suggesting repression still occurs in the absence of these fragments. In contrast, the *Δprox3.0* exhibits a small increase in expression, suggesting it removes some repression, and the *Δprox4.4* has the highest increase in expression, suggesting it removes the most repression. Furthermore, since the *Δprox1.3* and *Δprox2.6* mutants do not display increased expression, but the *Δprox3.0* mutant (which overlaps the *Δprox2.6* completely and *Δprox1.3* partially) does, this suggests that there might be redundant repressive activity within the *Δprox1.3* and *Δprox2.6* fragments. Additionally, since the difference between the *Δprox3.0* and *Δprox2.6* deletions removes Sna binding (Fig. 1a, see local Sna ChIP peak), it is possible that the increased expression associated with *Δprox3.0* and *Δprox4.4* is due to a reduction in negative autoregulation.

### Negative autoregulation of the *sna* gene is mediated by the proximal enhancer

It has been demonstrated, both in *Drosophila* as well as in the mouse, that *sna* undergoes autoregulation (Peiró et al. 2006; Boettiger and Levine 2013). Autoregulation serves to buffer against over- or under-production of a gene product. This leads to only small differences in total transcriptional output between the presence of 1 copy of a gene and 2 copies (Boettiger and Levine 2013; Murugan and Kreiman 2022). Conversely, when a gene is not autoregulated, transcription from 1 copy of the gene is expected to be half the amount as 2 copies.

To test whether *sna* autoregulation is disrupted in the mutants, we measured the relative levels of transcription at 2 copies (sna*/sna*) or 1 copy (sna*/*Df*), with the asterisk symbolizing individual mutant backgrounds. To ensure the levels could be compared between mutant genotypes, and to avoid any confounding influences associated with variable maternal effects, we used the transheterozygous crosses with the deficiency as the mother. As expected, the expression level of the 1 copy condition in the wild type (*yw*) reaches nearly the same levels as the 2 copies condition (1 to 2 copies ratio is 0.98; Fig. 3a). We also tested the *sna*^*1*^ mutant, which contains a premature stop codon and removes 4 zinc fingers, preventing DNA binding (Hemavathy, Meng, and Ip 1997). Autoregulation should not occur in *sna*^*1*^ and 1 copy is hypothesized to produce half, or 0.5 times, the transcripts as 2 copies. For *sna*^*1*^, we found the ratio of 1 to 2 copies to be 0.52, reflecting a lack of autoregulation as predicted (Fig. 3a).

Having confirmed the assumptions of our assay with wild type and *sna*^*1*^, we then compared the ratio of 1 to 2 copies for the enhancer mutations. We used Tukey's HSD for multiple comparisons after performing 1-way ANOVA to determine whether the 1 and 2 copies levels were statistically different. If autoregulatory feedback is broken, this statistical test would unambiguously determine whether there is a difference in levels between 1 and 2 copies. The majority of the mutants did not show a significant difference in 1 to 2 copy ratios, and none of them had a ratio of 0.5, which would signify a complete loss of autoregulation (Fig. 3a, Supplementary Fig. 3a in Supplementary File 1). The *Δprox4.4* ratio of 0.76, however, was significantly different, suggesting that autoregulation is partially lost in the *Δprox4.4* (Fig. 3a). The other genotype with increased expression, the *Δprox3.0* (Fig. 2k), also exhibited a minor effect, with a ratio of 0.82, but since the difference between 1 and 2 copies was not significant, this suggested that autoregulation is retained (Fig. 3a). The levels in the *Δdist0.4*, the region of Sna binding in the distal, were also not significantly different between 1 and 2 copies, but appear to decrease at 1 copy (Supplementary Fig. 3a in Supplementary File 1).

Upon comparing the compensation we observed with previously published values, the 1 to 2 copies ratio of 0.98 for *yw* was greater than the 0.78 ratio that was reported (Boettiger and Levine 2013). The deficiency used for the 1 copy assay, chosen based on its use in previous studies (Ashraf et al. 1999; Boettiger and Levine 2013) removes ∼32 genes, several of which are known to be involved in oogenesis. Therefore, we also tested a subset of lines for a maternal effect and found that changing the direction of the cross (i.e. using *Df(2L)Osp29* males) resulted in the wild type 1 to 2 copies ratio decreasing from 0.98 to 0.73 and the *Δprox4.4* decreasing from 0.76 to 0.5 (Supplementary Fig. 4a in Supplementary File 1 compared with Fig. 3a). These results indicate that the deficiency line has a maternal effect on *sna* levels leading to across the board increases in 1 copy levels. Regardless of the maternal contribution, the *Δprox4.4* exhibits increased *sna* expression as well as a loss of compensation at 1 copy (Figs. 2k and 3a, and Supplementary Fig. 4a in Supplementary File 1). In contrast, none of the distal deletions have an increase in expression, including the region bound by Sna in ChIP-seq assays (*Δdist0.4*; Figs. 1a and 2k). This suggests that negative autoregulatory feedback occurs via sequences associated with the proximal enhancer, not the distal enhancer.

### Increasing activator-binding affinity can affect autoregulation, but not in the intact locus

To provide insight into the mechanism by which the 4.4 kb proximal enhancer region mediates negative autoregulation, we focused on the region within this enhancer occupied by Sna. We noticed that Sna-, Dl-, and Twi-binding data identified by ChIP-seq appear to be located in a similar part of the proximal enhancer (Fig. 1a; see also Fig. 3b). This observation led us to hypothesize that perhaps Sna negative autoregulatory feedback acts to locally inactivate Dl, potentially by competing for binding or preventing histone displacement.

We hypothesized that by disrupting the balance of Sna to Dl, we might increase *sna* levels. To achieve this, 4 low-affinity Dl-binding sites (Ip et al. 1992) were mutated into sites of higher affinity (*dl L > H*, Fig. 3c), which we predicted might allow Dl to outcompete Sna's ability to locally inactivate it. However, the expression levels and the dosage compensation (ratio of 0.89) in *dl L > H* were similar to wild type (Fig. 3a, d, and d′), indicating that either the levels of Dl already saturate the low-affinity sites or the mutation did not increase the activation sufficiently to overcome negative autoregulation. Since our deletion analysis indicated that there was redundant repressive activity in the *Δprox1.3* and *Δprox2.6* regions (discussed above), we attempted to sensitize the assay by simultaneously mutating the Dl-binding sites and removing the *Δprox2.6* element (*Δprox2.6_dl L > H*; Fig. 3b). Unlike either the *Δprox2.6* or *dl L > H* mutations individually, the *Δprox2.6_dl L > H* double mutant leads to enhanced *sna* expression and a decrease in compensation at 1 copy (1 to 2 copy ratio of 0.73; Fig. 3a, e, and e′). This result suggests that saturating Dl levels is not sufficient to explain the observed lack of increase in the *dl L > H*, and that there is a more complicated interaction between Dl and Sna. For instance, the increased Dl affinity may only affect the binding of Sna in close proximity, such that the loss of autoregulation is only uncovered when also removing the more distant Sna-binding sites in the *Δprox2.6*.

### Modeling provides support for the view that the proximal element mediates negative autoregulation of *sna*

**Fig. 4.**
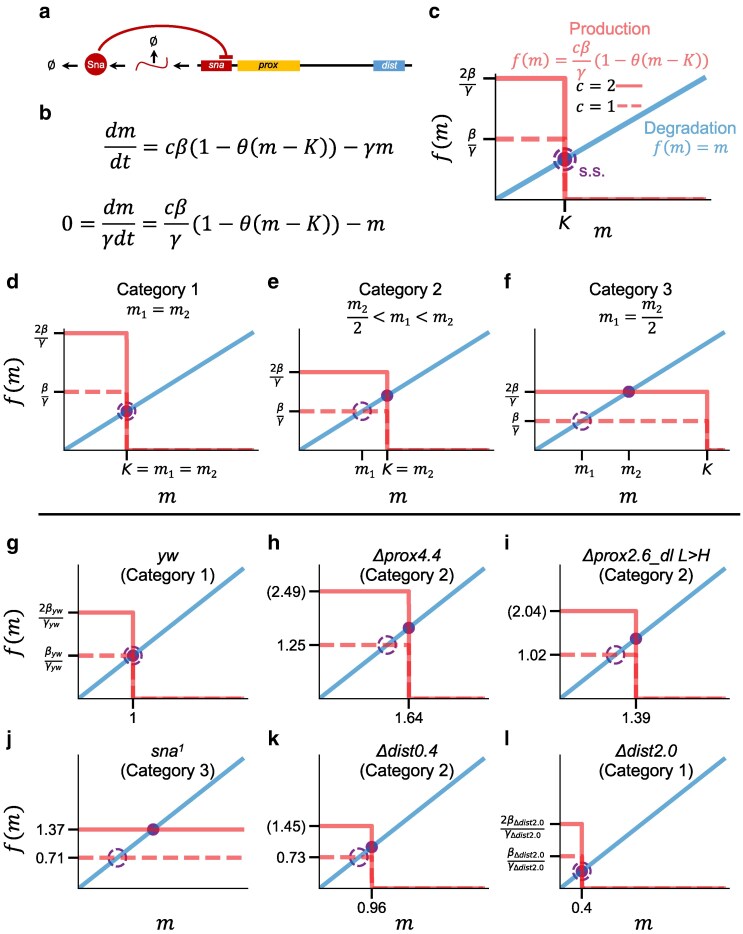
Modeling provides support for the view that the proximal element mediates negative autoregulation of *sna.* a) A model of negative autoregulation, where the protein product represses transcription. b) A differential equation that describes negative autoregulation assuming that the regulation is proportional to the concentration of *sna* mRNA (*m*), and that the Hill coefficient (*n*) goes to infinity. *θ* represents the Heaviside function, also called a step function. *β* is the production rate of *sna* mRNA, *c* is the copy number, *γ* is the degradation rate of *sna* mRNA, and *K* is the threshold of repression. c) A plot demonstrating the solution to the equation in b when solved at steady state. The red line represents the production component, which is the step function. The straight blue line represents the degradation component. The steady state (s.s., purple) is where the blue and red line cross. The steady state for 2 copies is shown with a solid purple circle and the steady state for 1 copy is shown as an open, dashed circle. The straight line (blue) can cross the step function (red) in 2 places, either at the horizontal line at *f*(*m*) = *cβ*/*γ* or the vertical line at *m* = *K*. The step function is represented as a solid line when *c* = 2 and a dotted line when *c* = 1. d–f) Similar plots as in *c*, except showing examples of the possible behaviors for the solutions for 1 copy and 2 copies. d) An example where the ratio of 1 to 2 copies is >0.9 and the steady state for both 1 copy and 2 copies is at *K* (category 1). e) An example where the ratio of 1 to 2 copies is between 0.6 and 0.9, the steady state for 2 copies is at *K*, and the steady state for 1 copy is at *β*/*γ* (category 2). f) An example where the ratio of 1 to 2 copies is <0.6, the steady state for 2 copies is at 2*β*/γ, and the steady state for 1 copy is at *β*/*γ* (category 3). g–l) Similar plots to c–f except for each genotype, with the category noted. The values of *cβ*/*γ* and *K* are displayed on the axes. The displayed numbers are found from the data listed in Supplementary Table 1 in Supplementary File S2. When the data are not known a variable name is displayed. If the data are inferred, such as the value of 2*β*/*γ*, the value is displayed in parentheses. In *sna*^*1*^, there is no repression, so there is no *K*.

In a system where the output is dependent on the coordination of 2 enhancers and multiple transcriptional inputs, including the product of the gene itself (rev. in Boettiger and Levine 2013; Irizarry and Stathopoulos 2021), interpretation of steady-state data can be difficult. To gain deeper insight into the key components that influence the expression of *sna* in the various mutant conditions and to test the hypothesis that the proximal element mediates negative autoregulation of *sna*, we generated a simple model using ODEs and mass action kinetics (Fig. 4a, Supplementary Fig. 5a and b in Supplementary File 1, see Supplementary Methods in Supplementary File 2 for further details).


(1) $$\begin{array}{c} \frac{dm}{dt}=c\beta \left(\frac{K^{n}}{m^{n}+K^{n}}\right)-\gamma m \end{array}$$

(2) $$\begin{array}{c} \frac{dm}{dt}=c\beta \left(\frac{1}{{\left(\frac{m}{K}\right)}^{n}+1}\right)-\gamma m \end{array}$$

This model can be written in 2 forms (Equations 1 and 2), which include a Hill function for modeling repression. *m* is the concentration of *sna* mRNA, *t* is time, and *c* is the copy number of the *sna* gene. The model also includes 4 parameters: *β* is the production rate of *sna mRNA*, *γ* is the degradation rate of *sna* mRNA, *n* is the Hill coefficient, and *K* is the concentration at which the production rate is at half its maximum value. *K* is also related to the dissociation constant of Sna binding, inversely related to binding affinity, and can be thought of as a threshold for repression.

The model was simplified to reduce the number of parameters, and the system was assumed to be at steady state (Equations S1 and S6–S8; see Supplementary Methods in Supplementary File 2). 1/*K* was solved analytically in terms of the Hill coefficient, *n*, and the ratio of 1 to 2 copies (Equations S9–S13; see Supplementary Methods in Supplementary File 2), and the solution was plotted (Supplementary Fig. 5c and d in Supplementary File 1). For the purposes of this simple model, we chose to consider all of the 1 to 2 copy ratios for mutant conditions without regard to statistical differences. To this end, we analytically solved for the mutant condition Hill coefficients in terms of the control Hill coefficient, the 1 to 2 copies ratio, and the ratio of the mutant conditions to the control (Equations S14–S18, Supplementary Methods in Supplementary File 2; Supplementary Fig. 5c and d in Supplementary File 1). For a defined set of parameters, we verified that the analytical solution gave relative levels that are equal to the observed data (Supplementary Fig. 5e in Supplementary File 1), except for in the case of *sna*^*1*^.

When comparing the levels of expression in *sna*^*1*^ to the observed data, the levels predicted by the model are >2 times higher than the observed data for both 1 copy and 2 copies (Equation S1 in Supplementary Methods in Supplementary File 2; Supplementary Fig. 5e in Supplementary File 1 compared with Fig. 3a). In addition, *sna*^*1*^ levels are lower than the *Δprox4.4* deletion. One possible explanation for these observations is that the premature stop codon in *sna*^*1*^ leads to nonsense mediated decay, which can result in degradation of the mRNA when a premature stop codon is detected during translation (Hemavathy, Meng, and Ip 1997; Kurosaki, Popp, and Maquat 2019). Thus, the measured levels of *sna*^*1*^ transcript might be lower because the degradation rate is higher. An alternative explanation is that *sna*^*1*^ disrupts a positive feedback mechanism when Sna is unable to bind DNA. This feedback could result from any net positive interaction, such as Sna-mediated activation of an activator or Sna-mediated repression of a repressor. A neurogenic repressor that represses *sna* and is repressed by Sna has been proposed in previous studies (Lagha et al. 2013). Both nonsense mediated decay of the mRNA transcript and disruption of a positive feedback loop are valid hypotheses, and future experimentation is needed to determine which is occurring in *sna*^*1*^.

While the model can describe the data (Supplementary Fig. 5e in Supplementary File 1), varying the Hill coefficient of the control gave different analytical solutions for the remaining parameters. This implies that infinite solutions are possible when only the relative levels are known. Nevertheless, this analytical solution (Supplementary Fig. 5 in Supplementary File 1) also suggests that cooperativity, represented by the Hill coefficient, *n*, is likely large. For the control, *yw*, the values for *n* ranged from 30 to infinity, and the *n* for all of the mutants except for *sna*^*1*^ were >2. To simplify and provide further interpretability of the model, we analyzed the system when the Hill coefficient*, n*, goes to infinity. This is an extreme for values of *n* (Supplementary Fig. 5c in Supplementary File 1) and results in a step function (Fig. 4b), that when displayed can visually illustrate behavior for 1 and 2 copy levels in various genetic backgrounds (Fig. 4c–f).


(3) $$\begin{array}{c} \frac{dm}{dt}=c\beta (1-\theta (m-K))-\gamma m \end{array}$$

(4) $$\begin{array}{c} 0=c\beta (1-\theta (m-K))-\gamma m \end{array}$$

(5) $$\begin{array}{c} 0=\frac{c\beta }{\gamma }(1-\theta (m-K))-m \end{array}$$

In these equations, *θ* represents the Heaviside function, or step function, and replaces the Hill function when *n* goes to infinity. Equation (3) can be solved at steady state to yield Equation (5) (Equations 3–5). In this equation, *K* can be thought of as the threshold for repression. If *m* is <*K*, expression is fully on, and if *m* is >*K* then expression is fully off. If *cβ*/*γ* is >*K*, then the concentration of *sna* (*m*) at steady state is equal to *K* (Fig. 4c). If *cβ*/*γ* is <*K*, then the concentration of *sna* (*m*) at steady state is equal to *cβ*/*γ*. Graphically, the steady state can be represented by the intersection of the step function (Fig. 4c, red) and the straight line *f(m) = m* (Fig. 4c, blue), since steady state occurs when these 2 functions equal each other (purple circles in Fig. 4c and Equation 5 when d*m*/d*t* = 0).

This analysis gives 3 categories of behaviors. For category 1, the ratio of the 1 to 2 copies is ≥0.9. In this case, the intersection of the step function and line is at *K* for both 1 and 2 copies (solid and dashed purple circle), and both the 1 and 2 copies conditions are undergoing repression (Fig. 4d). For category 2, the 1 to 2 copies ratio is between 0.6 and 0.9. In this second case, the 2 copies condition intersects at *K* (solid purple circle) and is undergoing repression, and the 1 copy condition intersects at *β*/*γ* (dashed purple circle) and is not undergoing repression (Fig. 4e). For category 3, the 1 to 2 copies ratio is ≤0.6. In this third case, the 2 copies condition intersects at 2*β*/*γ* (solid purple circle), the 1 copy condition intersects at *β*/*γ* (dashed purple circle), and both the 1 and 2 copies conditions are not undergoing repression (Fig. 4f).

This analysis provides relative estimates of *K* or *β*/*γ* for the various mutant conditions, as all of the measurements were normalized by the levels of the control at 2 copies (Fig. 3a). An increase in *K* can be interpreted as an increase in the threshold for repression, which means that the concentration of Sna needs to be higher for it to repress. On the other hand, a decrease in the net production rate of 1 or 2 copies (*β*/*γ* or 2*β*/*γ*) likely reflects a change in the *sna* mRNA production rate (*β*), as the degradation rate of mRNA (*γ*) should not be affected by deletions of any enhancer region.

When the control, *yw*, was analyzed using this categorization, it was assigned to category 1 as its 1 to 2 copies ratio of 0.98 is >0.9 (Fig. 4d). Thus, the steady-state value for 2 copies of *yw* (*m*_2_) equals the steady-state value for 1 copy (*m*_1_), and both are equal to *K* (Fig. 4g). The only conclusion about *β*/*γ* that can be drawn is that it must be >*K*. If it was <*K*, the steady state of 1 copy would occur at *β*/*γ* instead of *K*. Since the levels were normalized by the value for 2 copies of the control, *K* for *yw* by definition is equal to 1.

We also used this categorization to understand the *sna*^*1*^ mutant. The *sna*^*1*^ mutant was assigned to category 3 as its 1 to 2 copies ratio of 0.52 is <0.6 (Figs. 3a and 4f and j). This means that the 2 copies level (*m*_2_) should be equal to 2*β*/*γ* and the 1 copy level (*m*_1_) should be equal to *β*/*γ*. If the *sna*^*1*^ mutation does not affect the production rate (*β*) or the degradation rate (*γ*), it is expected that *β*/*γ* in *sna*^*1*^ would equal *β*/*γ* in *yw*. However, the 1 copy levels of *sna*^*1*^ are 0.71 when normalized by the 2 copies *yw* levels. Since 0.71 is <1, the minimum *β*/*γ* for *yw* as described in the preceding paragraph, this indicates that the *sna*^*1*^*β*/*γ* is less than the *yw β*/*γ* (Fig. 4g). This deviation from the model prediction provides further support that *sna*^*1*^ levels are lower than expected, as described above for the analytical solution.

In addition to *yw* and *sna*^*1*^, we analyzed and categorized the mutant states, represented by their 1 to 2 copies ratio and levels relative to *yw*, using the graphical steady-state analysis (Fig. 4g–i, Supplementary Fig. 6 in Supplementary File 1). The majority of the mutants were assigned to category 2, with ratios ranging from 0.6 to 0.9, except for the *Δprox2.6* and the *Δdist2.0.* When analyzed using the step function, the *Δprox4.4*, *Δprox2.6_dl L > H*, and the *Δprox3.0* had increased values for *K*, representing increased repression thresholds, compared to *yw* (Fig. 4h and i and Supplementary Fig. 6b in Supplementary File 1 compared with Fig. 4g). The relative values of *K* indicate that the change in repression threshold was greatest in the *Δprox4.4*, suggesting that the *Δprox2.6_dl L > H* and the *Δprox3.0* mutants still retain some functional sites for repression that are removed in the *Δprox4.4*.

The *Δdist2.0* was assigned to category 1, as the 1 to 2 copies ratio was >0.9. In contrast to the *Δprox4.4*, *Δprox2.6_dl L > H*, and *Δprox3.0*, the *Δdist2.0* had a reduction in *K*, which implies that the concentration of Sna needed to repress is lower (Fig. 4l, Supplementary Table 1 in Supplementary File 2). One explanation for this phenomenon is that the simple model treats repression from both the proximal and distal sites as a single parameter. By removing the less effective distal-binding sites, the remaining strong proximal sites could potentially appear stronger. Alternatively, this might indicate that the Sna-binding sites in the distal enhancer can act as a biological sink, locally depleting the available Sna protein and limiting its binding to the proximal enhancer. Thus, removing the proximal enhancer, or altering the ability of Sna to bind to it, leads to lower repressive activity, while removing the distal leads to increased repressive activity.

In the *Δdist0.4* mutant, the 2 copies level was similar to *yw*, while the *Δdist0.4* 1 copy level was decreased. The *Δdist0.4* mutant was assigned to category 2, as its 1 to 2 copies ratio was 0.75 (Supplementary Fig. 3a in Supplementary File 1). However, how negative autoregulatory feedback might support a lower 1 copy level is difficult to understand intuitively, so the graphical steady-state analysis was applied to provide insight. From this analysis and categorization, *K* remains relatively unchanged for the *Δdist0.4*, but *β*/*γ* is reduced in the *Δdist0.4* compared with *yw* (Fig. 4k). The estimated *β*/*γ* for the *Δdist0.4* is 0.73, which is <1, the minimum value for the *yw β*/*γ* (Fig. 4k). As the *Δdist0.4* is not expected to change the degradation rate (*γ*), the production rate (*β*) is predicted to vary between the *Δdist0.4* and *yw*. One interpretation of this reduction in the production rate is that activator-binding sites are removed in the *Δdist0.4* deletion (see Conclusion for further discussion). Taken together, this modeling approach supports 2 hypotheses: that the proximal element mediates negative autoregulatory feedback, and that the *Δdist0.4* fragment likely contributes to activation/production of Sna.

### Mathematical modeling does not support a distance-only effect on *sna* expression

Since deletion of the proximal element also moves the distal element closer to the promoter, we sought to explore how distance affects the simple negative autoregulatory model. Under conditions without autoregulatory feedback, the ratio of 1 to 2 copies is always 1 half (Equations S19–S23 in Supplementary Methods in Supplementary File 2). Since ratios vary from 0.73 to 0.98, this implies that some feedback must occur in this system when Sna is produced (see Fig. 3a). To explore how a change in distance might affect a model including feedback, we used the same approach as mentioned above, assuming that the Hill coefficient, *n*, goes to infinity (Fig. 4), and modified this model to account for a distance-related effect that results in the distal element being closer to the promoter when the proximal element is removed (Supplementary Fig. 7 in Supplementary File 1).

Three conditions were tested: when only repression is reduced (Supplementary Fig. 7b in Supplementary File 1), when only a distance effect is added (Supplementary Fig. 7c in Supplementary File 1), and when both repression is reduced and a distance effect is added (Supplementary Fig. 7d in Supplementary File 1). To model a distance effect, *β* was increased, since moving the enhancer closer to the promoter is expected to increase the maximum level of expression (i.e. when no negative autoregulation occurs). However, when *β* alone was increased, meaning only a distance effect was added, there was no effect on the intersection occurring at *m = K* (Supplementary Fig. 7c′ in Supplementary File 1), even if *β* is further increased (Supplementary Fig. 7c″ in Supplementary File 1). In contrast, changing the *K* to mimic a reduction in repression does change where the intersection occurs (Supplementary Fig. 7b′ in Supplementary File 1). When both *β* and *K* are changed, different outcomes are possible depending on the magnitude of the changes (Supplementary Fig. 7d′ and d″ in Supplementary File 1). While this analysis cannot provide molecular insights, it does suggest that a reduction in repression is necessary to explain the observed data, as a distance effect alone is not sufficient.

### Limitations of the study

Our study suggests Sna exhibits strong cooperativity. To test the impact of this assumption, we solved the model when the Hill coefficient, *n*, goes to infinity as well as when *n* is equal to 4. We found that the error between assuming *n* goes to infinity vs *n* equals 4 is relatively low, both for the estimate of *K* and the estimate of *β*/*γ* (Supplementary Fig. 8g–i in Supplementary File 1). Previous studies, however, modeled Sna binding and cooperativity in an enhancer of *rho*, finding that Sna had weak cooperativity in this context (Sayal et al. 2016). The findings of both our study and the previous study are not necessarily contradictory. While Sna-mediated repression of *rho* leads to complete silencing of *rho* in the presumptive mesoderm (Kosman et al. 1991), Sna-mediated repression of itself does not completely silence *sna* output, but instead contributes to precise regulation of *sna* expression. Furthermore, Sna repressing its own transcription is a unique scenario, making it possible that this autoregulatory feedback requires different cooperativity than when Sna acts to repress other target genes.

Additionally, Sayal *et al*. modeled Sna action in the context of Dl and Twi binding, whereas our approach assumes that transcription factor binding of Sna to DNA is independent of other factors. One can imagine that Dl, Twi, or other factors that were not included in the model, might compete with or otherwise alter Sna's ability to bind to DNA. By using Hill functions, our approach is able to model repression without assuming a molecular mechanism for how Sna binds enhancers. This can be useful when a mechanism is not known. However, use of Hill functions to model repression is limited in elucidating the principles underlying a phenomenon.

This modeling approach also consisted of a number of other assumptions. Firstly, we chose to model the system agnostic to whether Sna binding at either the proximal or distal enhancer could influence the production rate either locally within the bound enhancer, or at a distance in the alternative enhancer. Although Sna is typically classified as a local repressor (Gray and Levine 1996), it can also act as an antilooping factor, affecting activator activity over longer distances (Chopra, Kong, and Levine 2012). Secondly, this modeling approach does not include all of the downstream targets of Sna and any potential feedback, which may play an important role in *sna* levels. For example, the interaction between Sna and Notch is not included (Cowden and Levine 2002; Ozdemir et al. 2014). Lastly, we also assumed that the system is at steady state and that the degradation rate of mRNA is constant. While it seems plausible that the system is at steady state, given the increased length of nc14, this assumption would be violated if degradation is not constant. In this scenario, we would expect our data to have increased variability arising from the difference in levels across the narrow time window we assayed (mid-to-late nc14). In addition, the model predictions would need to be refined to include nonconstant degradation.

### Conclusion

**Fig. 5.**
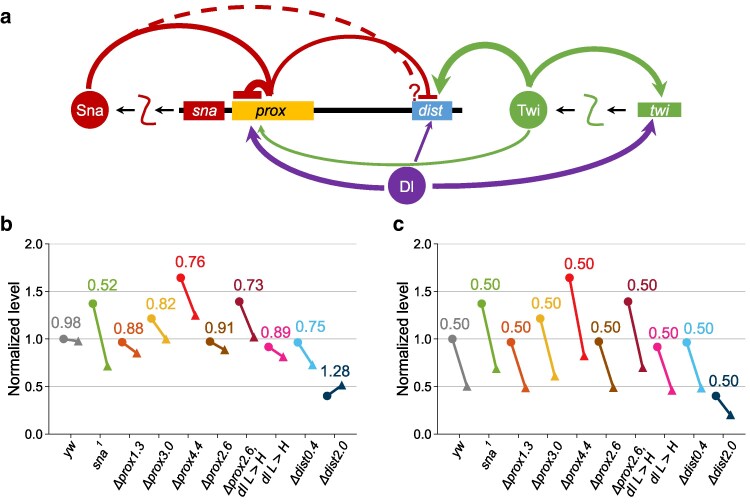
Model of how *sna* is regulated, including by negative autoregulatory feedback. a) Schematic of *sna* regulation, including key activators Dorsal (Dl), Twist (Twi), as well as Sna autoregulatory feedback at the proximal (prox), and distal (dist) elements. The question mark represents the unknown function of Sna binding in the distal element. b) The normalized mean *sna* levels replotted from Fig. 3a for key genotypes. c) The hypothesized *sna* levels when 1 copy levels are expected to be 0.50 times 2 copy levels if dosage compensation or feedback does not occur. For the graphs in b and c, the circle represents the *sna* levels for 2 copies, and the triangle represents the *sna* levels for 1 copy. The pairs of data are connected by a solid line with the numeric ratio of 1 copy over 2 copies displayed above. For each plot, the values are all normalized by dividing by the steady-state value of the control, *yw*, in the 2 copies condition.

In summary, we utilized mutational analysis and mathematical modeling to provide evidence that the *sna* locus undergoes negative autoregulation mediated through the proximal enhancer. We propose that autoregulation acting through the proximal enhancer sets the level of *sna* expression by balancing the strong activating function of the distal enhancer (Fig. 5a). Our data provide evidence for autoregulation of *sna* transcription, as the 1 to 2 copies ratio for various conditions is not 0.5 as would be expect without feedback (Fig. 5b and c). Furthermore, the modeling provides support for the hypothesis that the proximal element mediates negative autoregulation, as the *Δprox4.4* and *Δprox2.6_dl L > H* had increased *K* values, or increased repression thresholds. In addition, the modeling also supports that a distance effect alone cannot explain the observed data and suggests that the levels in the *Δdist0.4* might involve other activating factors.

These predictions from the modeling provide a foundation for future studies to expand upon. One area in particular that merits further study is understanding how the *Δdist0.4* results in decreased 1 copy levels. The difference between 1 vs 2 copies of *Δdist0.4* is likely not due to a loss of negative autoregulation, as the *Δdist0.4* did not result in higher levels compared with *yw* in the 2 copies condition. In addition, the modeling suggests that the difference was due to a lower *β*. However, removing this genomic region might have multiple effects on *sna* transcriptional regulation. For instance, the deleted region exhibits binding for the transcriptional activator and pioneer factor Zelda (Zld), in addition to Sna (Fig. 1a; Koenecke et al. 2016) and also affects at least one Dl binding site (Dl3, Syed et al. 2023). Loss of activation input may be epistatic to loss of Sna repression input, or Sna input at the distal may directly support activation. Previous studies have shown Sna can also act as a context-dependent activator (Rembold et al. 2014). In light of this fact, it is possible that Sna itself may contribute to activation in this context of the distal CRM. Future studies will be needed to differentiate between the hypotheses that this element is important for the binding of activators or is responsible for positive autoregulation.

This prediction that Sna autoactivation occurs in the distal element introduces an intriguing idea: autoregulation of the *sna* gene may function differently through 2 enhancers—negatively through the proximal enhancer and positively through the distal enhancer. This kind of regulation has been observed in other systems, such as the Runt transcription factor influencing *sloppy paired 1* (Hang and Gergen 2017). Systems involving both positive and negative autoregulation are thought to provide finely tuned control of gene expression, allowing rapid response times while maintaining stability within a specific range (Gao and Stock 2018). Precise regulation of not only *sna* but mesoderm gene levels may be crucial to support gastrulation movements and require both negative and positive autoregulatory feedback. Our previous study showed that the expression levels of many mesoderm genes plateau preceding gastrulation (Sandler and Stathopoulos 2016).

In addition to helping determine Sna levels for specification of the mesoderm, previous studies have shown that the proximal enhancer of the *sna* gene is conditionally necessary for robust expression and survival at high temperature (Perry et al. 2010; Dunipace, Ozdemir, and Stathopoulos 2011), and here we show that this enhancer also mediates negative autoregulation. This raises the question of whether autoregulation is essential for development in stable laboratory conditions or becomes critical in environments with fluctuating temperatures, especially for *Drosophila*, which cannot regulate their body temperature. Furthermore, autoregulatory control is common among transcription factors, being associated with over half of those studied in humans, and is important across various species (Kiełbasa and Vingron 2008; Minchington, Griffiths-Jones, and Papalopulu 2020). Understanding how these autoregulatory mechanisms function and when they are necessary, even if conditionally required, could have significant implications for our knowledge of gene regulation.

## Supplementary Material

iyaf058_Supplementary_Data

## Data Availability

*Drosophila* strains and other reagents generated in this study will be available upon request from the lead contact. A github repository with the code for quantitative and statistical analyses was generated and is publicly available: https://github.com/StathopoulosLab/Dunipace_2024. Additional code used to verify image segmentation is available: https://github.com/StathopoulosLab/McGehee_2024. Any additional information required to reanalyze the data shown in this paper is available from the lead contact upon request. ChiP-Seq datasets were previously published and can be accessed using https://doi.org/10.1186/s13059-016-1057-2 GEO accession numbers: Sna: GSM1689688; toll10b_twi_1: GSM1689698; toll10b_dl_1: GSM1689690 (Koenecke et al. 2016, Edgar et al. 2002). Supplemental material available at GENETICS online.

## References

[iyaf058-B1] Alon U . 2007. Network motifs: theory and experimental approaches. Nat Rev Genet. 8(6):450–461. doi:10.1038/nrg2102. 17510665

[iyaf058-B2] Ashraf SI , Hu X, Roote J, Ip YT. 1999. The mesoderm determinant snail collaborates with related zinc-finger proteins to control Drosophila neurogenesis. EMBO J. 18(22):6426–6438. doi:10.1093/emboj/18.22.6426. 10562554 PMC1171705

[iyaf058-B3] Boettiger AN , Levine M. 2013. Rapid transcription fosters coordinate snail expression in the Drosophila embryo. Cell Rep. 3(1):8–15. doi:10.1016/j.celrep.2012.12.015. 23352665 PMC4257496

[iyaf058-B4] Bothma JP , Garcia HG, Ng S, Perry MW, Gregor T, Levine M. 2015. Enhancer additivity and non-additivity are determined by enhancer strength in the Drosophila embryo. eLife. 4:e07956. doi:10.7554/eLife.07956. 26267217 PMC4532966

[iyaf058-B5] Carver EA , Jiang R, Lan Y, Oram KF, Gridley T. 2001. The mouse snail gene encodes a key regulator of the epithelial-mesenchymal transition. Mol Cell Biol. 21(23):8184–8188. doi:10.1128/MCB.21.23.8184-8188.2001. 11689706 PMC99982

[iyaf058-B6] Choi HMT , Schwarzkopf M, Fornace ME, Acharya A, Artavanis G, Stegmaier J, Cunha A, Pierce NA. 2018. Third-generation hybridization chain reaction: multiplexed, quantitative, sensitive, versatile, robust. Development. 145(12):dev165753. doi:10.1242/dev.165753. PMC6031405 29945988

[iyaf058-B7] Chopra VS , Kong N, Levine M. 2012. Transcriptional repression via antilooping in the Drosophila embryo. Proc Natl Acad Sci U S A. 109(24):9460–9464. doi:10.1073/pnas.1102625108. 22645339 PMC3386088

[iyaf058-B8] Cowden J , Levine M. 2002. The snail repressor positions notch signaling in the Drosophila embryo. Development. 129(7):1785–1793. doi:10.1242/dev.129.7.1785. 11923213

[iyaf058-B9] Crews S . 2017. Creating cell type-specific mutants by enhancer mutagenesis. Genes Dev. 31(7):629–631. doi:10.1101/gad.299586.117. 28446593 PMC5411702

[iyaf058-B10] Crews ST , Pearson JC. 2009. Transcriptional autoregulation in development. Curr Biol. 19(6):R241–R246. doi:10.1016/j.cub.2009.01.015. 19321138 PMC2718735

[iyaf058-B11] Dunipace L , Ozdemir A, Stathopoulos A. 2011. Complex interactions between cis-regulatory modules in native conformation are critical for Drosophila snail expression. Development. 138(18):4075–4084. doi:10.1242/dev.069146. 21813571 PMC3160101

[iyaf058-B101] Dunipace L, Saunders A, Ashe HL, Stathoupoulos, A. 2013. Autoregulatory feedback controls sequential action of cis-regulatory modules at the brinker locus. Dev Cell. 26 (5): 536–543. doi:10.1016/j.devcel.2013.08.010

[iyaf058-B102] Edgar R, Domrachev M, Lash AE. 2002. Gene expression omnibus: NCBI gene expression and hybridization array data repository. Nucl Acids Res. 30(1):207–210. 10.1093/nar/30.1.207 PMC99122 11752295

[iyaf058-B12] Fischer MD , Graham P, Pick L. 2024. The ftz upstream element drives late ftz stripes but is not required for regulation of ftz target genes. Dev Biol. 505:141–147. doi:10.1016/j.ydbio.2023.11.004. 37977522 PMC10843599

[iyaf058-B13] Galindo MI , Fernández-Garza D, Phillips R, Couso JP. 2011. Control of distal-less expression in the Drosophila appendages by functional 3′ enhancers. Dev Biol. 353(2):396–410. doi:10.1016/j.ydbio.2011.02.005. 21320482 PMC3940868

[iyaf058-B14] Gao R , Stock AM. 2018. Overcoming the cost of positive autoregulation by accelerating the response with a coupled negative feedback. Cell Rep. 24(11):3061–71.e6. doi:10.1016/j.celrep.2018.08.023. 30208328 PMC6194859

[iyaf058-B15] Garcia M , Nahmad M, Reeves GT, Stathopoulos A. 2013. Size-dependent regulation of dorsal-ventral patterning in the early Drosophila embryo. Dev Biol. 381(1):286–299. doi:10.1016/j.ydbio.2013.06.020. 23800450 PMC3968923

[iyaf058-B16] Gratz SJ , Ukken FP, Rubinstein CD, Thiede G, Donohue LK, Cummings AM, O’Connor-Giles KM. 2014. Highly specific and efficient CRISPR/cas9-catalyzed homology-directed repair in Drosophila. Genetics. 196(4):961–971. doi:10.1534/genetics.113.160713. 24478335 PMC3982687

[iyaf058-B17] Grau Y , Carteret C, Simpson P. 1984. Mutations and chromosomal rearrangements affecting the expression of snail, a gene involved in embryonic patterning in DROSOPHILA MELANOGASTER. Genetics. 108(2):347–360. doi:10.1093/genetics/108.2.347. 17246230 PMC1202410

[iyaf058-B18] Gray S, Levine M. 1996. Short-range transcriptional repression in the Drosophila embryo. Genes Dev. 10(6):700–710. doi:10.1101/gad.10.6.700

[iyaf058-B19] Hang S , Gergen JP. 2017. Different modes of enhancer-specific regulation by runt and even-skipped during Drosophila segmentation. Mol Biol Cell. 28(5):681–691. doi:10.1091/mbc.E16-09-0630. 28077616 PMC5328626

[iyaf058-B20] Hemavathy K , Meng X, Ip YT. 1997. Differential regulation of gastrulation and neuroectodermal gene expression by snail in the Drosophila embryo. Development. 124(19):3683–3691. doi:10.1242/dev.124.19.3683. 9367424

[iyaf058-B21] Ip YT , Park RE, Kosman D, Yazdanbakhsh K, Levine M. 1992. Dorsal-twist interactions establish snail expression in the presumptive mesoderm of the Drosophila embryo. Genes Dev. 6(8):1518–1530. doi:10.1101/gad.6.8.1518. 1644293

[iyaf058-B22] Irizarry J , Stathopoulos A. 2021. Dynamic patterning by morphogens illuminated by cis-regulatory studies. Development. 148(2):dev196113. doi:10.1242/dev.196113. PMC7847269 33472851

[iyaf058-B23] Jiang J , Levine M. 1993. Binding affinities and cooperative interactions with bHLH activators delimit threshold responses to the dorsal gradient morphogen. Cell. 72(5):741–752. doi:10.1016/0092-8674(93)90402-C. 8453668

[iyaf058-B24] Kiełbasa SM , Vingron M. 2008. Transcriptional autoregulatory loops are highly conserved in vertebrate evolution. PLoS One. 3(9):e3210. doi:10.1371/journal.pone.0003210. 18791639 PMC2527657

[iyaf058-B25] Koenecke N , Johnston J, Gaertner B, Natarajan M, Zeitlinger J. 2016. Genome-wide identification of Drosophila dorso-ventral enhancers by differential histone acetylation analysis. Genome Biol. 17(1):196. doi:10.1186/s13059-016-1057-2. 27678375 PMC5037609

[iyaf058-B26] Kosman D , Ip YT, Levine M, Arora K. 1991. Establishment of the mesoderm-neuroectoderm boundary in the Drosophila embryo. Science. 254(5028):118–122. doi:10.1126/science.1925551. 1925551

[iyaf058-B27] Kurosaki T , Popp MW, Maquat LE. 2019. Quality and quantity control of gene expression by nonsense-mediated mRNA decay. Nat Rev Mol Cell Biol. 20(7):406–420. doi:10.1038/s41580-019-0126-2. 30992545 PMC6855384

[iyaf058-B28] Lagha M , Bothma JP, Esposito E, Ng S, Stefanik L, Tsui C, Johnston J, Chen K, Gilmour DS, Zeitlinger J, et al 2013. Paused pol II coordinates tissue morphogenesis in the Drosophila embryo. Cell. 153(5):976–987. doi:10.1016/j.cell.2013.04.045. 23706736 PMC4257494

[iyaf058-B29] Lamouille S , Xu J, Derynck R. 2014. Molecular mechanisms of epithelial-mesenchymal transition. Nat Rev Mol Cell Biol. 15(3):178–196. doi:10.1038/nrm3758. 24556840 PMC4240281

[iyaf058-B30] Leptin M , Grunewald B. 1990. Cell shape changes during gastrulation in Drosophila. Development. 110(1):73–84. doi:10.1242/dev.110.1.73. 2081472

[iyaf058-B31] Lou L , Bergson C, McGinnis W. 1995. Deformed expression in the Drosophila central nervous system is controlled by an autoactivated intronic enhancer. Nucleic Acids Res. 23(17):3481–3487. doi:10.1093/nar/23.17.3481. 7567459 PMC307227

[iyaf058-B32] Mauhin V , Lutz Y, Dennefeld C, Alberga A. 1993. Definition of the DNA-binding site repertoire for the Drosophila transcription factor SNAIL. Nucleic Acids Res. 21(17):3951–3957. doi:10.1093/nar/21.17.3951. 8371971 PMC309975

[iyaf058-B33] McGinnis W , Jack T, Chadwick R, Regulski M, Bergson C, McGinnis N, Kuziora MA. 1990. Establishment and maintenance of position-specific expression of the Drosophila homeotic selector gene deformed. Adv Genet. 27:363–402. doi:10.1016/S0065-2660(08)60030-9. 1971987

[iyaf058-B34] Minchington TG , Griffiths-Jones S, Papalopulu N. 2020. Dynamical gene regulatory networks are tuned by transcriptional autoregulation with microRNA feedback. Sci Rep. 10(1):12960. doi:10.1038/s41598-020-69791-5. 32737375 PMC7395740

[iyaf058-B35] Muqbil I , Wu J, Aboukameel A, Mohammad RM, Azmi AS. 2014. Snail nuclear transport: the gateways regulating epithelial-to-mesenchymal transition? Semin Cancer Biol. 27:39–45. doi:10.1016/j.semcancer.2014.06.003. 24954011 PMC4165636

[iyaf058-B36] Murugan R , Kreiman G. 2022. Multiple transcription auto regulatory loops can act as robust oscillators and decision-making motifs. Comput Struct Biotechnol J. 20:5115–5135. doi:10.1016/j.csbj.2022.08.065. 36187915 PMC9493064

[iyaf058-B37] Nüsslein-Volhard C , Wieschaus E, Kluding H. 1984. Mutations affecting the pattern of the larval cuticle in Drosophila melanogaster: I. Zygotic loci on the second chromosome. Wilhelm Roux's Archives of Developmental Biology. 193(5):267–282. doi:10.1007/BF00848156. 28305337

[iyaf058-B38] Ozdemir A , Ma L, White KP, Stathopoulos A. 2014. Su(H)-mediated repression positions gene boundaries along the dorsal-ventral axis of Drosophila embryos. Dev Cell. 31(1):100–113. doi:10.1016/j.devcel.2014.08.005. 25313963 PMC4201238

[iyaf058-B39] Park KW , Hong J-W. 2012. Mesodermal repression of single-minded in Drosophila embryo is mediated by a cluster of snail-binding sites proximal to the early promoter. BMB Rep. 45(10):577–582. doi:10.5483/BMBRep.2012.45.10.105. 23101512

[iyaf058-B40] Peiró S , Escrivà M, Puig I, Barberà MJ, Dave N, Herranz N, Larriba MJ, Takkunen M, Francí C, Muñoz A, et al 2006. Snail1 transcriptional repressor binds to its own promoter and controls its expression. Nucleic Acids Res. 34(7):2077–2084. doi:10.1093/nar/gkl141. 16617148 PMC1440880

[iyaf058-B41] Perry MW , Boettiger AN, Bothma JP, Levine M. 2010. Shadow enhancers foster robustness of Drosophila gastrulation. Curr Biol. 20(17):1562–1567. doi:10.1016/j.cub.2010.07.043. 20797865 PMC4257487

[iyaf058-B103] Port F, Bullock SL. 2016. Augmenting CRISPR applications in Drosophila with tRNA-flanked sgRNAs. Nat Methods. 13(10):852-854. doi:10.1038/nmeth.3972. Epub 2016 Sep 5. PubMed 27595403. PMC5215823 27595403

[iyaf058-B42] Port F , Chen H-M, Lee T, Bullock SL. 2014. Optimized CRISPR/cas tools for efficient germline and somatic genome engineering in Drosophila. Proc Natl Acad Sci U S A. 111(29):E2967–E2976. doi:10.1073/pnas.1405500111. 25002478 PMC4115528

[iyaf058-B104] Rauluseviciute I, Riudavets-Puig R, Blanc-Mathieu R, Castro-Mondragon JA, Ferenc K, Kumar V, Lemma RB, Lucas J, Chèneby J, Baranasic D , *et al*. 2024. JASPAR 2024: 20th anniversary of the open-access database of transcription factor binding profiles. Nucl Acids Res. 52(D1):D174–D182. doi:10.1093/nar/gkad1059 PMC10767809 37962376

[iyaf058-B43] Rembold M , Ciglar L, Yáñez-Cuna JO, Zinzen RP, Girardot C, Jain A, Welte MA, Stark A, Leptin M, Furlong EEM. 2014. A conserved role for snail as a potentiator of active transcription. Genes Dev. 28(2):167–181. doi:10.1101/gad.230953.113. 24402316 PMC3909790

[iyaf058-B44] Rockwell AL , Beaver I, Hongay CF. 2019. A direct and simple method to assess Drosophila melanogaster's viability from embryo to adult. J Vis Exp. (150) doi:10.3791/59996. 31524863

[iyaf058-B45] Sandler JE , Stathopoulos A. 2016. Quantitative single-embryo profile of Drosophila genome activation and the dorsal-ventral patterning network. Genetics. 202(4):1575–1584. doi:10.1534/genetics.116.186783. 26896327 PMC4905543

[iyaf058-B46] Sayal R , Dresch JM, Pushel I, Taylor BR, Arnosti DN. 2016. Quantitative perturbation-based analysis of gene expression predicts enhancer activity in early Drosophila embryo. eLife. 5:e08445. doi:10.7554/eLife.08445. 27152947 PMC4859806

[iyaf058-B47] Sharpe JL , Morgan J, Nisbet N, Campbell K, Casali A. 2023. Modelling cancer metastasis in *Drosophila melanogaster*. Cells. 12(5):677. doi:10.3390/cells12050677. 36899813 PMC10000390

[iyaf058-B48] Simpson P . 1983. Maternal-zygotic gene interactions during formation of the dorsoventral pattern in Drosophila embryos. Genetics. 105(3):615–632. doi:10.1093/genetics/105.3.615. 17246169 PMC1202177

[iyaf058-B201] Syed S , Duan Y, Lim B. 2023. Modulation of protein-DNA binding reveals mechanisms of spatiotemporal gene control in early *Drosophila* embryos. Elife. 12:e85997. doi:10.7554/eLife.85997. 37934571 PMC10629816

[iyaf058-B49] Tran HD , Luitel K, Kim M, Zhang K, Longmore GD, Tran DD. 2014. Transient SNAIL1 expression is necessary for metastatic competence in breast cancer. Cancer Res. 74(21):6330–6340. doi:10.1158/0008-5472.CAN-14-0923. 25164016 PMC4925010

[iyaf058-B50] Yokoshi M , Segawa K, Fukaya T. 2020. Visualizing the role of boundary elements in enhancer-promoter communication. Mol Cell. 78(2):224–35.e5. doi:10.1016/j.molcel.2020.02.007. 32109364

[iyaf058-B51] Zhao J , Perkins ML, Norstad M, Garcia HG. 2023. A bistable autoregulatory module in the developing embryo commits cells to binary expression fates. Curr Biol. 33(14):2851–64.e11. doi:10.1016/j.cub.2023.06.060. 37453424 PMC10428078

