# Supplementary material for: The proximal enhancer of the snail gene mediates negative autoregulatory feedback in Drosophila melanogaster
Source: Genetics. 2025 Mar 27;230(2):iyaf058. doi: 10.1093/genetics/iyaf058 (PMC12135202; doi:10.1093/genetics/iyaf058)
Supplement: iyaf058_Supplementary_Data [file iyaf058_supplementary_data.zip › Dunipace_FileS2_SupInfo.pdf]

SUPPLEMENTARY INFORMATION

1. **Supplementary Methods**
2. **Table S1:** Relative mean levels of *sna* for one and two copies conditions in various genotypes from data displayed in Figure 3a and Figure S3 in File S1; used to solve for *m* (concentration of *sna* mRNA) at steady state - see Figure 4.
3. **Table S2:** Fly stocks
4. **Table S3:** Primer Sequences

SUPPLEMENTARY METHODS

CRISPR-Cas9 Mediated Genome Modification:

To target deletions and mutations of various sizes to the distal part of the proximal enhancer (i.e. *Δprox3.0, Δprox2.6, Δprox2.6_dl L>H, dl L>H*), a transgenic line was generated expressing two guide RNAs (gRNAs) targeting the flanking region described as the proximal enhancer (Dunipace, Ozdemir, and Stathopoulos 2011). First, the unique PAM recognition sites were identified flanking this region using the flyCRISPR optimal target finder (https://flycrispr.org/target-finder). To delete the full proximal enhancer, two flanking regions that are located outside of the identified proximal enhancer (Dunipace, Ozdemir, and Stathopoulos 2011) were targeted due to limited availability of PAM sequences, and subsequently led to the generation of a 4.4kB deletion. These two sites were cloned into the plasmid pCFD4 (Addgene #49411). The plasmid was injected into *y2cho2v1; P {nos-phiC31\int.NLS}6X; attP2 (III)* (NIG-Fly #TBX-0003), generating the stable fly line pCFD4_Δp4.4. Integration in the genome at this position was confirmed by PCR/sequencing.

To generate the proximal deletion lines (*Δprox3.0, Δprox2.6, Δprox2.6_dl L>H, dl L>H*), Homology-Directed Repair (HDR)/CRISPR-Cas9 was used (Port et al. 2014; Gratz et al. 2014). A donor construct was generated using pHD-DsRed vector (Addgene plasmid #51434). Homology arms of approximately 1kb upstream and downstream of the region, were cloned with SmaI/NheI or AscI/XhoI, respectively. For the *dl L>H* and *dl Δprox2.6_L>H* mutations, a fragment containing the *dl* mutations was synthesized by Genscript and then was fused to the adjacent homology region (either the full genomic region or the truncated *Δprox2.6* homology arm) using overlap PCR before subcloning into the right arm of the pHD-DsRed vector. The changes to the Dl binding sites were evaluated by running the modified sequence through binding-site prediction software (Jaspar; Castro-Mondragon et al. 2022), which did not predict an effect on Sna binding. The *Δprox2.6, dl L>H,* and *Δprox2.6_dl L>H* mutations were made independently using the common gRNA line, pCFD4_Δp4.4, with specific, distinct HDR constructs to create each mutant line.

y2cho2v1;sp/CyO;P {nos-Cas9,y+,v +} 2A (NIG-Fly #Cas-0004) virgin flies were crossed with gRNA transgenic male flies. Embryos were collected and injected with 300 ng/μl of the donor vector. The deletion lines were screened by DsRed expression in adult fly eyes and sequencing. The DsRed marker was removed by crossing with a Cre expressing fly line *(y[1] w[67c23] P{y[+mDint2]=Crey}1b; D[*]/TM3, Sb[1],* BDSC #851). Crispr/Cas9 generated deletions/mutations were confirmed by PCRing the region of interest and subsequently sequencing.

gRNA plasmids for all other deletions were designed in the same manner and cloned into either pCFD4 or pCFD5 (Addgene #73914), but were directly injected into the

Cas9 expressing line (BDSC#51324: *w[1118]; PBac{y[+mDint2] GFP[E.3xP3]=vas-Cas9}VK00027*). The *Δprox4.4, Δdist2.0, and Δdist1.8* were generated without a DSRed homology repair template. HDR repair templates for *Δprox1.3 and Δdist0.4* were made as described above and co-injected along with the gRNA plasmid into BDSC#51324. For lines with DSRed, the marker was removed by crossing to the Cre expressing line. The sequences after Cre-mediated DsRed marker excision are provided below.

Sequences of mutations:

Uppercase: genomic sequence

Lowercase: sequence added after HDR or NHEJ

// marking deletion positions with no insertions

Underlined sequence marking Dl binding sites that were mutated, mutated bases lower case

>*Δprox1.3*

ATTGTCAACTTGAACAAATGAGCCAGGGAACAAGGTGCAAAAATGcatatgcacacctgcg
atcgtagtgccccaactggggtaacctttgagttctctcagttgggggcgtagataacttcgtataatgtatgctatacgaagtt
atagaagagcactagtaaagatctccatgcataaggcgcgcctaggCCCTGAGCTATGTTTTCCTAGCT
CTCATCGATCGCTGCCAAT

>*Δprox3.0*

CGACAAAGGATGTGACTCAGcggccgcggacatatgcacacctgcgatcgtagtgccccaactggggtaa
cctttgagttctctcagttgggggcgtagataacttcgtataatgtatgctatacgaagttatagaagagcactagtATGAC
CCACCAGGTAGGATG

>Δprox2.6

AGTCGACAAAGGATGTGACTCAGcggccgcggacatatgcacacctgcgatcgtagtgccccaactggg
gtaacctttgagttctctcagttgggggcgtagataacttcgtataatgtatgctatacgaagttatagaagagcactagtTC
TCAGCAAAAATTGACAAGAACAACAA

>dl L>H

TCCTCTTCGAACAATGTCAGTCGAGCTCTGTAGATCCCTGTGTTCCCTCTTCATTGT
CAACTTGAACAAATGAGCCAGGGAACAAGGTGCAAAAATGatgcacacctgcgatcgtagtgc
cccaactggggtaacctttgagttctctcagttgggggcgtagataacttcgTCTCAGCAAAAATTGACAAGA
ACAACAACAATGTCTATGGAAAATCGAACTTCATCCCAGCACCTGCAGAAATCCCG
AGCGAGTCGGGGAAAAAGTATTTAACCCCCGAAAGGGTTTTCCCCAAAATAATGAA
GTAATGAATGAAG<u>GGGTTTTcCc</u>TGGCCGCCAATCTACCTAATACTAATGAGC<u>gggT</u>
<u>TTTccc</u>GACCAGGAATTTTTGCAAGTCAGGTACTTCAACGGATATATGGGTTCGACA
AGTGCGGATTTTCCCGCGACATCAATGAGGACTTGGCCGGGTTATCCGCGGTGCT
CATCGGGCAATTCCGCGGCCGAGGACTTCATCGTAGTGATCATTAGGTAGATATGT
GCATGGATGTGACATGGCGATCATTGCGCGGAATAACACACGTAATAACCGAGAT
ATCCGGG<u>TtTTcccGcc</u>AGGTAGGATGTGAGGACATATAGAAAACCCCCAGCCAGTT
TTTCCACTCGTCGTGGCTTGTTTTGCTTGAGTTTCGCTGACTGCGTAATTGGATAAG
AT<u>gggTTTtCCc</u>GTTAAATCCTTCGCTGATCCACATCCGGACATTCGTCGAAGGAAAA

TCCATTGCAgggTTTtCcgGAATGGAAATGCGGCTGGGTTATTGGCTCGACATTTCCC
ATCTTCCCTCACGCCATTGGTTGCAGGATCGCGGGGAATTGGAATTCCGCGCTGG
AATTTTTTGTCACCTCTTGGGTTTATCAAAACTTTTGGGTTTGCTATGGATTTTTTCC
AATTTTACCACCGCGCCTGGTTTTTTTTT

>Δprox2.6_dl L>H

CGACAGCCTCCAGCCGGGCAATGAAATTCAAAAATTCAAAATCAAAGGGCAGGCC
TGAGTCGACAAAGGATGTGACTCAatgcacacctgcgatcgtagtgccccaactggggtaacctttgagtt
ctctcagttgggggcgtagataacttcgTCTCAGCAAAAATTGACAAGAACAACAACAATGTCTAT
GGAAAATCGAACTTCATCCCAGCACCTGCAGAAATCCCGAGCGAGTCGGGGAAAA
AGTATTTAACCCCCGAAAGGGTTTTCCCCAAAATAATGAAGTAATGAATGAAGGGG
TTTTcCcTGGCCGCCAATCTACCTAATACTAATGAGCgggTTTTcccGACCAGGAATTT
TTGCAAGTCAGGTACTTCAACGGATATATGGGTTCGACAAGTGCGGATTTTCCCGC
GACATCAATGAGGACTTGGCCGGGTTATCCGCGGTGCTCATCGGGCAATTCCGCG
GCCGAGGACTTCATCGTAGTGATCATTAGGTAGATATGTGCATGGATGTGACATGG
CGATCATTGCGCGGAATAACACACGTAATAACCGAGATATCCGGGTtTTcccGccAG
GTAGGATGTGAGGACATATAGAAAACCCCCAGCCAGTTTTTCCACTCGTCGTGGCT
TGTTTTGCTTGAGTTTCGCTGACTGCGTAATTGGATAAGAtgggTTTtCCcGTTAAATC
CTTCGCTGATCCACATCCGGACATTCGTCGAAGGAAAATCCATTGCAgggTTTtCcgG
AATGGAAATGCGGCTGGGTTATTGGCTCGACATTTCCCATCTTCCCTCACGCCATT
GGTTGCAGGATCGCGGGGAATTGGAATTCCGCGCTGGAATTTTTTGTCACCTCTTG
GGTTTATCAAAACTTTTGGGTTTGCTATGGATTTTTTCCAATTTTACCACCGCGCCT
GGTTTTTTTTT

>*Δdist2.0*

ATTCCAACATTTTGCTGTGTAGCACCCTTGAACTTGTTGTGAaTAAGAGGGCCAATT
GCATTTGTTCCTTCTTCCATTA

> *Δdist1.8*

TATATTTTTAAATTCCAACATTTTGCTGTGTAGCACCCTTGAACTTGTTGTGAACTCA
G//ACCGGACACTTGACTCTATGCGCCGGCGCAGCATCGCAGGTAGTCATCTGGAA
CAGGTAAACATTTGCCAATATCTC

>*Δdis0.4*

TCCTTGTAGGCGGCGTTTTCCCACGATTTTATAACTGGGCTCGACTTCCCATCTTC
CCACCGTCCATGTGgcggccgcggacatatgcacacctgcgatcataacttcgtataatgtatgctatacgaagt
tatagaagagcactagtaaagatctccatgcataaggcgcgccAATCCGTGTTAAATTGTTTGAAATTT
AATAAATTGGTTCCTTTGCAAAAACAGCTTAAATGGAAATACTG

>*Δprox4.4*

TCAAAATCAAAGGGCAGGCCagagtcgacaaaggatgtgacgcAGGGTGCGCCTGCGTCTG
CTCTTCC

Embryonic viability assays:

Embryonic viability assays were performed by placing 2 virgin females and 2 young males in a collection chamber with a yeasted grape juice plate. The flies were aged for 24 to 48 hours and then the grape juice plate was replaced and the flies were allowed to lay

overnight. The next day the number of embryos was counted and the plate was stored in a humidified chamber for 48 hours. The number of unhatched embryos at the end of the incubation time was counted and the viability was determined as the difference between unhatched and total embryos divided by total number of embryos. The *Δdist2.0 and Δprox1.3_Δdist2.0* lines were assayed using a GFP marked balancer (BDSC 36320). Only the non-GFP, homozygous mutant, embryo counts are reported here. All viability assays were done with a minimum of three biological replicates.

Image quantification:

To quantify the levels of *sna* in an individual embryo, functions in MATLAB were developed. First, the average projection across 20 z-stacks was generated for each image. To segment the entire embryo, the DAPI signal was used and a gaussian blur of three was performed. The blurred image was then thresholded at a normalized threshold of 0.02 to generate a mask. The mask was then morphologically opened with a disk of radius ten, holes were filled, and objects under 1000 pixels were removed. To segment the signal, the *sna* channel was processed in a similar way, with a gaussian blur of three, a normalized threshold of 0.005, a radius of five for morphological opening, and area of 1000 pixels for object detection. Properties for both the entire embryo and the *sna* segmented domain were obtained using MATLAB's regionprops, including area, minimum and maximum Feret properties, the centroid, the major and minor axis of a fit ellipse, orientation of the fit ellipse, and mean intensity. Any *sna* signal that was detected outside of the embryo was removed. To calculate the background levels, first the *sna* signal mask was removed from the embryo mask to give a background mask. Then the

mean intensity was calculated in the *sna* channel using the background mask. In addition the area was also calculated. All lengths and areas were converted to microns. Finally, to normalize the mean intensity, the mean intensity of the background signal was subtracted from the mean intensity of the *sna* signal. The area, minimum Feret distance, and maximum Feret distance were normalized by dividing by the same metric for the embryo mask. Segmentation was manually checked by overlaying the mask on the image using a custom MATLAB function. The individual data was plotted as well as the mean ± SD between embryos. Data was normalized to the control, by dividing all data by the mean of the control, *yw*. Data was pooled across multiple experiments by grouping the control normalized data.

Modeling:

Simple negative autoregulation:

To generate a model of the system we used mass-action kinetics, where rates are proportional to constants and the concentration of species. Using these principles we generated a series of Ordinary Differential Equations (ODEs). We modeled repression of *sna* using a Hill function, which limits the output from zero to one. This type of modeling for activation and repression does not include interactions between the various activators and repressors. Instead, activation and repression are dependent on the concentration of the activator or repressor, the concentration at which the production rate is half of its maximum (denoted *K* with an accompanying subscript) and the Hill coefficient, which is a measure of the cooperativity of binding. For example, if Sna binding is cooperative, this cooperativity is between Sna protein, not other proteins such as Dl or Twi. The Hill

function is then multiplied by a constant *β*, which summarizes the maximum activation possible when no repressor is present. We also include *c* which is the *sna* copy number. In the Hill function, *n* is the Hill coefficient, and *K* is the concentration of Sna at which the production rate is half of its maximum. We also include a degradation term that includes a constant degradation rate, γ_{sna}, and is proportional to the concentration of *sna* mRNA. While we assume the degradation rate is constant, it is possible that it is not. If this were true, it would violate our assumption that the system is at steady-state (see below). In this model, the repression mediated by Sna is summarized by having the concentration of Sna be proportional to *sna* mRNA (denoted as *m*). This is a reasonable assumption as explicitly modeling Sna protein results in Sna concentration being proportional to *sna* mRNA concentration. The ODE to model simple negative autoregulation is shown in eq. 1 and 2. Since these equations include four parameters for which values have not been measured, they were nondimensionalized to reduce the number of parameters. Nondimensionalization forces the steady state to be *c* for *sna* when no autoregulation occurs, regardless of production rates and degradation rates:

$$\frac{d\widehat{m}}{d\hat{t}} = c\left(\frac{1}{\left(\frac{\widehat{m}}{K}\right)^{n} + 1}\right) - \widehat{m} \tag{S1}$$

In this equation $\widehat{m}$ is the nondimensionalized *sna* mRNA concentration. In the nondimensionalized equations there are only two parameters, *K* and the Hill Coefficient, *n*. t_o, m_o, $\widehat{m}$, and $\hat{t}$ are defined as follows

$$t_o = \frac{1}{\gamma} \tag{S2}$$

$$m_o = \frac{\beta}{\gamma} \tag{S3}$$

$$\widehat{m} = \frac{m}{m_o} \tag{S4}$$

$$\hat{t} = \frac{t}{t_o} \tag{S5}$$

It was assumed that the measured levels of *sna* mRNA are at steady-state. If this assumption is not true, then the dynamics of *sna* concentration over time are needed to properly fit the model to the data. When eq. 6 is solved for steady-state it yields:

$$0 = c\left(\frac{1}{\left(\frac{\widehat{m}_{ss}}{K}\right)^n + 1}\right) - \widehat{m}_{ss} \tag{S6}$$

$$\widehat{m}_{ss} = c\left(\frac{1}{\left(\frac{\widehat{m}_{ss}}{K}\right)^n + 1}\right) \tag{S7}$$

$$0 = \left(\frac{1}{K}\right)^n \widehat{m}_{ss}{}^{(n+1)} + \widehat{m}_{ss} - c \tag{S8}$$

This can be converted in to terms of the ratio of one to two copies and *n* and solved for 1/*K* to give

$$r = \frac{\widehat{m}_1}{\widehat{m}_2} = \frac{m_1}{m_2} \tag{S9}$$

$$\frac{1}{K} = \left(\frac{2 - \widehat{m}_2}{\widehat{m}_2{}^{(n+1)}}\right)^{\frac{1}{n}} \tag{S10}$$

$$\widehat{m}_2 = \frac{2r^{(n+1)} - 1}{r^{(n+1)} - r} \tag{S11}$$

$$\frac{1}{K} = \left(\frac{2 - \left(\frac{2r^{(n+1)} - 1}{r^{(n+1)} - r} \right)}{\left(\frac{2r^{(n+1)} - 1}{r^{(n+1)} - r} \right)^{(n+1)}} \right)^{\frac{1}{n}} \tag{S12}$$

$$\frac{1}{K} = \left(r^{(n+1)} - r\right) \left(\frac{1 - 2r}{(2r^{(n+1)} - 1)^{(n+1)}} \right)^{\frac{1}{n}} \tag{S13}$$

Equation 14 is the ratio, r, of mRNA levels when the *sna* levels at one copy are divided by the *sna* levels at two copies, and $\widehat{m}_2$ is the concentration of *sna* transcripts at two copies. $1/K$ can be expressed in terms of $\widehat{m}_2$ and n (eq. 15), and $\widehat{m}_2$ can be expressed in terms of the ratio of one to two copies and n (eq. 16). When eq. 16 is substituted into eq 15, it yields eq. 17 which can be simplified for eq. 18. The ratio calculated from the data was used to plot the values of $1/K$ and n that correspond to that ratio (Figure 4b,c). To determine how the mutant parameters were related to the control parameters and the the ratio of the mutant levels to the control levels, the Hill coefficient for a mutant (n_m) was analytically solved in terms of the Hill coefficient for the control (n_c), ratios of the mutant one copy levels to two copies levels (r_m), the control one copy levels to two copies levels (r_c), and the ratio (R) of mutant two copies levels ($\widehat{m}_{2m}$) to control two copies levels ($\widehat{m}_{2c}$).

$$\widehat{m}_{2_m} = \frac{2{r_m}^{(n_m+1)} - 1}{{r_m}^{(n_m+1)} - r_m} \tag{S14}$$

$$\widehat{m}_{2_c} = \frac{2{r_c}^{(n_c+1)} - 1}{{r_c}^{(n_c+1)} - r_c} \tag{S15}$$

$$R = \frac{\widehat{m}_{2_m}}{\widehat{m}_{2_c}} \tag{S16}$$

$$n_m = \frac{\ln\left(\frac{1 - R r_m \widehat{m}_{2_c}}{2 - R\widehat{m}_{2_c}}\right)}{\ln(r_m)} - 1 \tag{S17}$$

$$n_m = \frac{\ln\left(\frac{1 - Rr_m\left(\frac{2r_c^{(n_c+1)} - 1}{r_c^{(n_c+1)} - r_c}\right)}{2 - R\left(\frac{2r_c^{(n_c+1)} - 1}{r_c^{(n_c+1)} - r_c}\right)}\right)}{\ln(r_m)} - 1 \qquad (S18)$$

Eq. 19 can be substituted into eq. 21 and then solved for n_m to yield eq. 22. Eq. 20 can then be substituted into eq. 22 to yield eq. 23. From this analysis, it becomes apparent that the mutant parameters can take on infinitely many values when only r_m, r_c, and R are known. $1/K$ for both the mutant and control can be calculated from eq. 18. In order to make this comparison, the *yw* and mutant data has to be nondimensionalized or normalized. We divided all conditions by the *yw sna* levels at two copies. This assumes that the conditions have the same nondimensionalization factor for m_o (eq. 8), since this is the only way the nondimensionalization factor would cancel when normalizing by *yw* levels. While this is convenient to allow comparison of the data to the model, it is unlikely that the production rates of transcripts would be equal between different enhancer mutations (eq. 8, β). However, this is still a useful exercise since it demonstrates that many parameter sets work.

For modeling sna^1, the repressive Hill equation in equation 3 was removed, or replaced with one, the equivalent to no autoregulation. There should be no autoregulation in a sna^1 mutant, since Sna has lost most of its zinc fingers and likely does not bind to DNA in sna^1 mutants (Hemavathy, Meng, and Ip 1997).

Analytical solution when no feedback occurs:

$$\frac{dm}{dt} = c\beta(A; P) - \gamma m \tag{S19}$$

$$\gamma m_{ss} = c\beta(A_{ss}; P) \tag{S20}$$

$$m_{ss} = \frac{c\beta(A_{ss}; P)}{\gamma} \tag{S21}$$

$$\frac{m_{ss_1}}{m_{ss_2}} = \frac{\beta(A_{ss}; P)\gamma^{-1}}{2\beta(A_{ss}; P)\gamma^{-1}} \tag{S22}$$

$$\frac{m_{ss_1}}{m_{ss_2}} = \frac{1}{2} \tag{S23}$$

If the production of *sna* mRNA (*m*) has no feedback from Sna protein (eq. 24), then regardless of the form the production rate takes ($c\beta(A; P)$), the ratio of the steady-state values (eq. 25, 26) of one to two copies (eq. 27) is always one half (eq. 28). Here *c* is the copy number, β is a function of the production rate, *A* is the concentration of some species that activates *sna* transcription, *P* is the set of parameters affecting activation, and γ is the degradation rate of *sna*. Since there is no autoregulatory feedback in this model, the details of *A*, *P*, $\beta(A; P)$, and γ do not matter as they will always simplify to one (eq. 27), leaving only the ratio of the copy number to determine the ratio of the mRNA levels.

Comparison of the simple negative autoregulation model when n *is four to when* n *goes to infinity:*

To compare the model when the Hill coefficient *n* is four to when *n* goes to infinity, eq. 1 and eq. 5 were solved for steady state for various *K* and β/γ. Eq. 1 was solved using Brent's method implemented by scipy.optimize.brentq to determine the roots when Eq.1 was set to zero to solve for steady state. When *n* goes to infinity, the steady state value

occurs at $m = K$ when $c\beta/\gamma$ is greater than or equal to K or at $m = c\beta/\gamma$ when $c\beta/\gamma$ is less than K. K and β/γ were varied from 0.01 to 3.0 and the steady state values were determined for both eq. 1 and eq. 5. The percentage difference between the two models' steady states at each K and β/γ was calculated by taking the absolute value of the difference between the steady states, dividing by the average of the steady states, and multiplying by one hundred (Figure S8a-g in File S1). In addition, over the same K and β/γ, the steady states that were found when n equals four, were used to do the same step function analysis as was done with the data. Specifically, if the one to two copies ratio was greater than 0.9 the mean of the one and two copies levels was used as an estimate of K, and β/γ was unknown. If the one to two copies ratio was between 0.6 and 0.9, then the two copies level was used as an estimate of K and the one copy level was used as an estimate of β/γ. If the one to two copies ratio was less than 0.6, then the average of the one copy level and the two copies level divided by two was used as an estimate of β/γ and K was unknown. The percentage error was then calculated between the known K and the estimate of K as well as the known β/γ and the estimate of β/γ. Percentage error was calculated by taking the absolute value of the difference between the known value and the estimate, dividing by the known value, and multiplying by one hundred. When plotted, the white domain is the area where either K or β/γ could not be estimated by the step function analysis (Figure S8h,i in File S1). From both of these analyses, the error between the models where n goes to infinity and n equals four is relatively low (Figure S8g-i in File S1).

Table S1: Relative mean levels of *sna* for the one and two copies conditions in various genotypes from data displayed in Figure 3a and Figure S3 in File S1; used to solve for m (concentration of *sna* mRNA) at steady state - see Figure 4.

Genotype	Two Copies Level (m_2)	One Copy Level (m_1)	Category	Relationship to K or β/γ
yw	1.00	0.98	Category 1	$K = m_2 = m_1$
sna^1	1.37	0.71	Category 3	$2\beta/\gamma = m_2$<br>$\beta/\gamma = m_1$
Δprox1.3	0.97	0.85	Category 1	$K = m_2 = m_1$
Δprox3.0	1.21	1.00	Category 1	$K = m_2 = m_1$
Δprox4.4	1.64	1.25	Category 2	$K = m_2$<br>$\beta/\gamma = m_1$
Δprox2.6	0.97	0.88	Category 1	$K = m_2 = m_1$
Δprox2.6, dl L>H	1.39	1.02	Category 2	$K = m_2$<br>$\beta/\gamma = m_1$
dl L>H	0.92	0.81	Category 1	$K = m_2 = m_1$
Δdist0.4	0.96	0.73	Category 2	$K = m_2$<br>$\beta/\gamma = m_1$
Δdist2.0	0.40	0.51	Category 1	$K = m_2 = m_1$

Table S2: Fly stocks

Fly stock	Source
Adh[n7] sna[1] cn[1] vg[1]/CyO	BDSC 25127
w[1118]; Df(2L)osp29, Adh[UF] osp[29] pr[1] cn[1]/CyO, P{ry[+t7.2]=sevRas1.V12}FK1	BDSC #3078, rebalanced with *CyO, P{HB-lacZ}GS1* (see below)
y2cho2v1; P {nos-phiC31\int.NLS}6X; attP2 (III)	NIG-Fly #TBX-0003
y2cho2v1;sp/CyO;P {nos-Cas9,y+,v +} 2A	NIG-Fly #Cas-0004
y[1] w[67c23]; sna[Sco]/CyO, P{w[+mC]=Crew}DH1	BDSC #1092
y[1] w[*]; wg[Sp-1]/CyO, P{HB-lacZ}GS1	This study, made from BDSC#6650 crossed to BDSC#59967
y[1] w[*]; wg[Sp-1]/CyO, P{w[+mC]=ActGFP}JMR1	This study, made from BDSC#3632 crossed to BDSC#59967
w[1118]; PBac{y[+mDint2] GFP[E.3xP3]=vas-Cas9}VK00027)	BDSC#51324
pCFD4_Δprox4.4	*This study*
Δprox1.3	*This study*
Δprox2.6	*This study*
Δprox3.0	*This study*
Δprox4.4	*This study*
Δdist0.4	*This study*
Δdist1.8	*This study*
Δdist2.0/CyO, P{HB-lacZ}GS1	*This study*
Δprox1.3_Δdist2.0/CyO, P{HB-lacZ}GS1	*This study*
Δprox2.6_dl L>H	*This study*
dl L>H	*This study*

Table S3: Primer sequences

Primer Sequence	Description
TATATAGGAAAGATATCCGGGTGAACTTCGACAAAGGATGTGACTCAGGTGTTTTAGAGCTAGAAATAGCAAG	gRNA delprox 4.4 F_pcfd4
ATTTTAACTTGCTATTTCTAGCTCTAAAACGGGAGGGTGCGCCTGCGTCTCGACGTTAAATTGAAAATAGGTC	gRNA delprox 4.4 R_pcfd4
GCGGCCCGGGTTCGATTCCCGGCCGATGCAAACAAGGTGCAAAAATGGGAGTTTTAGAGCTAGAAATAGCAAG	gRNA delprox 1.3 F_pcfd5
ATTTTAACTTGCTATTTCTAGCTCTAAAACATGCCCTGAGCTATGTTTTCTGCACCAGCCGGGAATCGAACCC	gRNA delprox 1.3 R_pcfd5
GCGGCCCGGGTTCGATTCCCGGCCGATGCATCCCACCGTCCATGTGTTGCGTTTTAGAGCTAGAAATAGCAAG	gRNA deldist0.4 F_pcfd5
ATTTTAACTTGCTATTTCTAGCTCTAAAACCTTATCGTTCTCCGTGTTAATGCACCAGCCGGGAATCGAACCC	gRNA deldist0.4 R_pcfd5
TATATAGGAAAGATATCCGGGTGAACTTCGAGAGTCAAGTGTCCGGTCGGGTTTTAGAGCTAGAAATAGCAAG	gRNA deldist1.8 F_pcfd4
ATTTTAACTTGCTATTTCTAGCTCTAAAACTGCCTGAGTTCACAACAAGTCGACGTTAAATTGAAAATAGGTC	gRNA deldist1.8 R_pcfd4
GCGGCCCGGGTTCGATTCCCGGCCGATGCACTTGAACTTGTTGTGAACTCGTTTTAGAGCTAGAAATAGCAAG	gRNA deldist2.0 F_pcfd5
ATTTTAACTTGCTATTTCTAGCTCTAAAACCTTATCGTTCTCCGTGTTAATGCACCAGCCGGGAATCGAACCC	gRNA deldist2.0 R_pcfd5
CATGCTAGCCTTGCGACAATAGAGCTTGCTG	delprox1.3 HDR LA-F
GTGCATATGCATTTTTGCACCTTGTTCC	delprox1.3 HDR LA-R
GGCGCGCCTAGGCCCTGAGCTATGTTTTCCTAG	delprox1.3 HDR RA-F
TAACTCGAGCTGCAGCGTTTCAGGGATAGATCCTG	delprox1.3 HDR RA-R
CATGCTAGCCTTGCGACAATAGAGCTTGCTG	dl L>H HDR LA-F

GTGCATATGCATTTTTGCACCTTGTTCC	dl L>H HDR LA-R
AGCACTAGTTCTCAGCAAAAATTGACAAG	dl L>H HDR RA-F
GGGCCTAGGCATAGGAGATGTCATTA	dl L>H HDR RA-R
GCAGCATGCGAAAAATCTTCCATTTGG	deldist0.4 LA-F
AATGCGGCCGCCACATGGACGGTGGGAAGATG	deldist0.4 LA-R
AATGGCGCGCCAATCCGTGTTAAATTGTTTGA	deldist0.4 RA-F
ATTCTCGAGCCGAAGTTGTCCAGCTG	deldist0.4 RA-R
CTA GCGGGCGCGCCC GGA ACC GGT TTC CAG AT	delprox RA-R
GTACGTACTAGTTCTCAGCAAAAATTGACAAGAA	del prox 2.6 RA-F
GTACGTACTAGTATGACCCACCAGGTAGGATG	del prox 3.0 RA-F
ATAAGCAGGACACACGCGATGTCCCCTAATCAGCTTAGTCGCCGTAGCATAATGGAACGTTGGGGGCAGC	del2.6 dl L>H left-f
TGCCCGATGAGCACCGCGGATAACCCGGCCAAGTCCTCATTGATGTCGCGGGAAAATCCGCACTTGTCGA	del2.6 L>H left-r
AGTCAGGTACTTCAACGGATATATGGGTTCGACAAGTGCGGATTTTCCCGCGACATCAATGAGGACTTGG	del2.6 L>H right-f
ACCTTCGCAGAGAGGTGTTCCAAAAATAGCACTGATAATGAGCCGAATTCCGAGACACCAAGTGTCAACTC	del2.6 L>H right-r
