# Supplementary material for: The proximal enhancer of the snail gene mediates negative autoregulatory feedback in Drosophila melanogaster
Source: Genetics. 2025 Mar 27;230(2):iyaf058. doi: 10.1093/genetics/iyaf058 (PMC12135202; doi:10.1093/genetics/iyaf058)
Supplement: iyaf058_Supplementary_Data [file iyaf058_supplementary_data.zip › File_S1_GENETICS-2025-307897.pdf]

SUPPLEMENTARY FIGURES

Figure S1

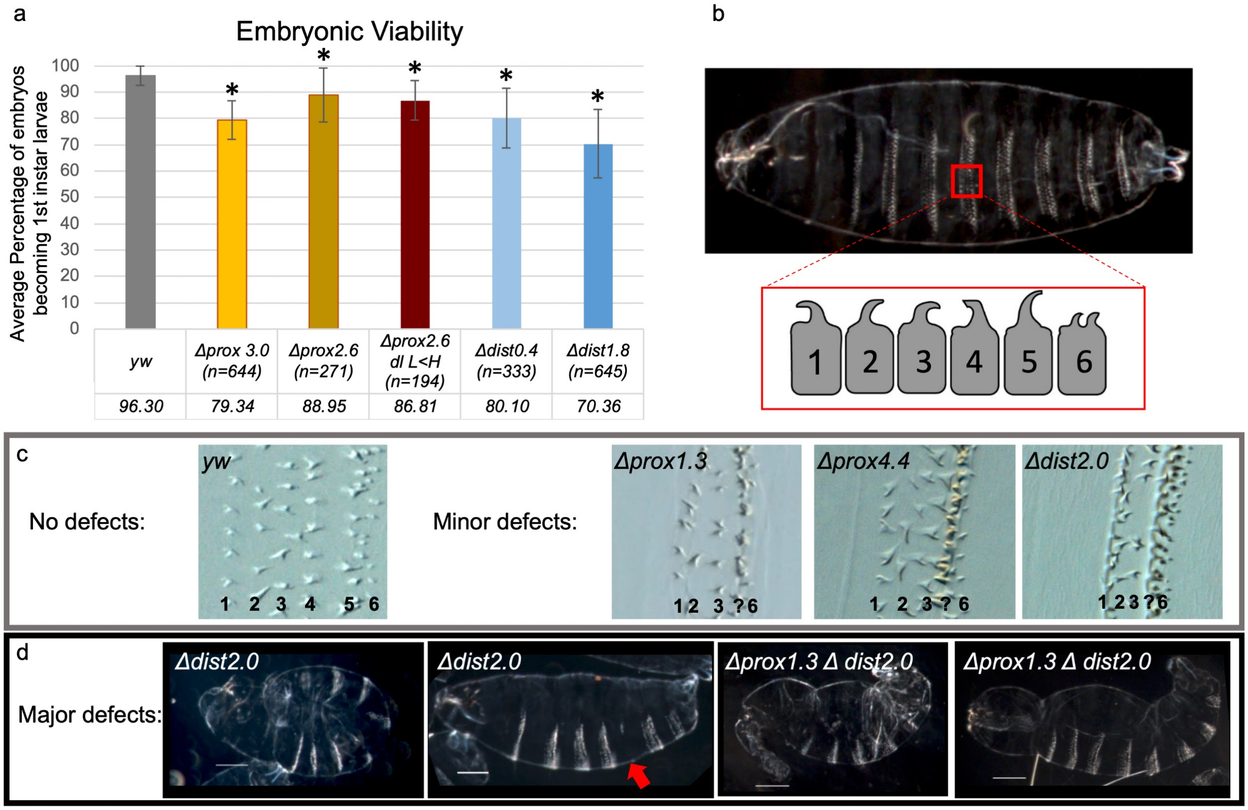

Figure S1: Viability is moderately reduced in enhancer deletions for portions of the proximal and distal. (a) Viability at 25°C shown for the lines not represented in Figure 1. Wildtype (*yw*) data is the same as in Figure 1, reproduced here for comparison. The asterisk represents p-values < 5.56×10^{-3} as computed through bootstrapping (see Materials and Methods). Total number of embryos counted (n) is shown under the genotype label, mean ± sd shown in black. (b) Phase contrast image of dorsal view of wildtype cuticle. Red box indicates the region of the denticle belts shown for each of the mutants in (c) along with a graphical representation of the shape of the bristles in each of the six rows in the denticle belt. **(c)** Representative images of what was scored as minor

defects in cuticles in Figure 1c. Magnified image of the center of a single denticle belt as indicated in (b). *yw* is shown for comparison to highlight changes seen in denticle band bristle organization. A general narrowing of the denticle band and a loss of bristles is seen in all of the mutants, as well as a loss of differentiation in the region of the fourth and fifth row of bristles. **(d)** Representative images of major defects in cuticles from Figure 1c, including curved embryos and missing denticle belts (red arrow).

Figure S2

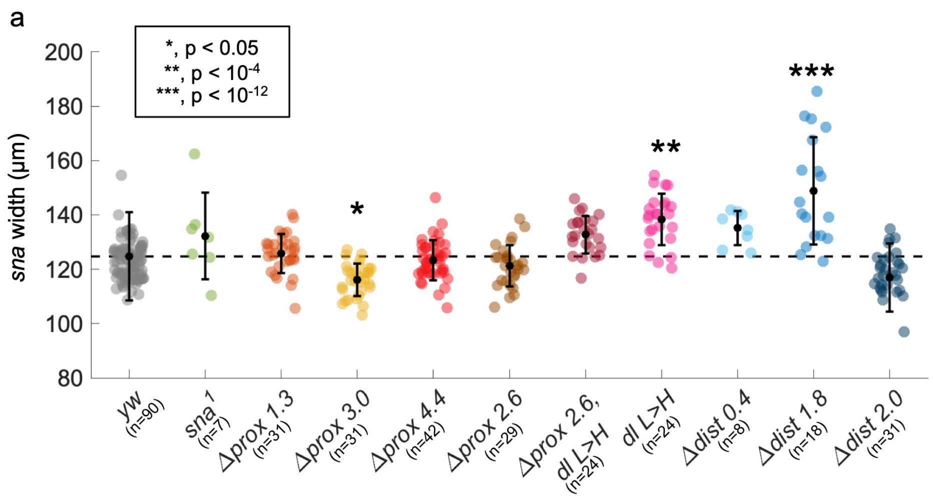

Figure S2: Range of widths exhibited by mutants compared to control. (a) The widths of *sna* expression in microns for the control (*yw*) and mutant conditions. The widths were determined by computing the minimum Feret, or caliper, diameter for the *sna* expression domain. To serve as a reference, the dashed line marks the mean of the control. One asterisk represents p-values < 0.05, two asterisks represents p-values < 10^{-4}, and three asterisks represents p-values < 10^{-12} when comparing the mutant conditions to the control using Tukey's HSD for multiple comparisons after performing one way ANOVA. Total number of embryos measured (n) is shown under the genotype label; mean ± sd is shown in black.

Figure S3

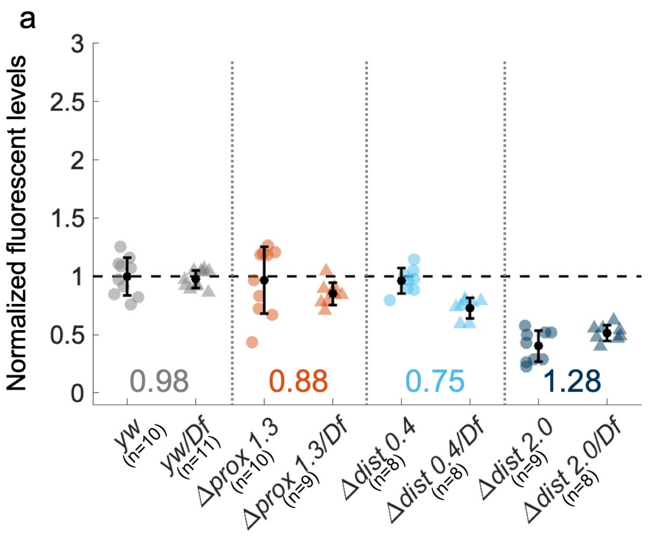

Figure S3: Negative autoregulation is not lost in the *Δprox1.3*, *Δdist0.4,* or *Δdist2.0.*

(a) Quantification of one and two copies for deletions not shown in Figure 3. Numbers displayed below paired conditions represent the levels of one copy divided by the levels of two copies. No means were significantly different when comparing one to two copies using Tukey's HSD for multiple comparisons after performing one way ANOVA. Total number of embryos measured (n) is shown under the genotype label, mean ± sd shown in black. Maternal genotype for all one copy crosses is *Df(2L)Osp29/CyO. yw* data is replotted from Figure 2 for comparison. The *Δprox1.3* reaches a similar level between the one and two copies, suggesting negative autoregulation is intact. The *sna* levels go down in the *Δdist0.4* at one copy, but do not go up compared to wildtype at two copies.

Figure S4

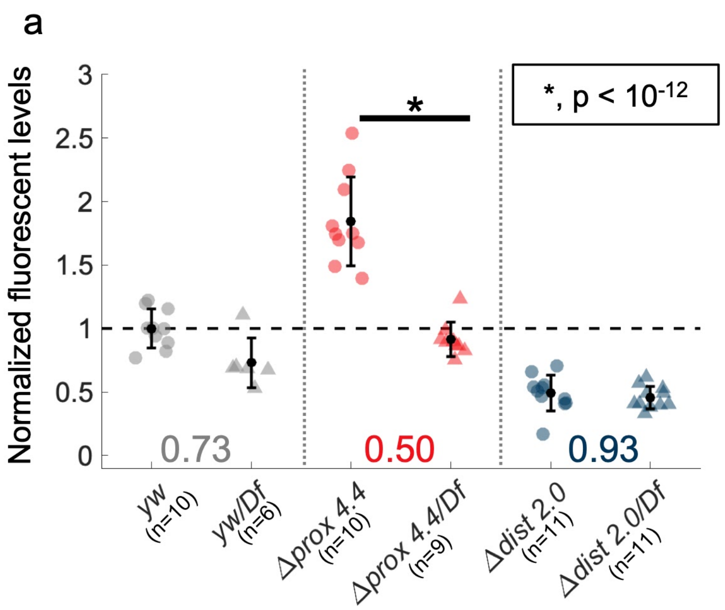

Figure S4: The one to two copies compensation is not as strong when controlled for maternal effects. (a) Quantification of *sna* levels when either two copies or one copy are present, performed as in Figure 2. Numbers displayed below paired conditions represent the levels of one copy (e.g. *yw/Df*, on the right) divided by the levels of two copies (e.g. *yw/yw,* on the left, which by convention is denoted *yw*). The asterisk represents p-values < 10^{-12} when comparing one to two copies using Tukey's HSD for multiple comparisons after performing one way ANOVA. Maternal genotype for all one copy crosses is the respective *yw* or *sna* CRISPR/Cas9 mutant genotype, with one copy crossed to *Df(2L)Osp29/CyO* males*.* In this scenario, with the cross flipped so the control or the mutation is the virgin and the deficiency is the male, autoregulatory compensation is not as great (compare with Figure 3a) and there is a bigger difference between one and two copies (i.e. for *yw* the ratio changes from 0.98 to 0.73). Total number of embryos measured (n) is shown under the genotype label.

Figure S5

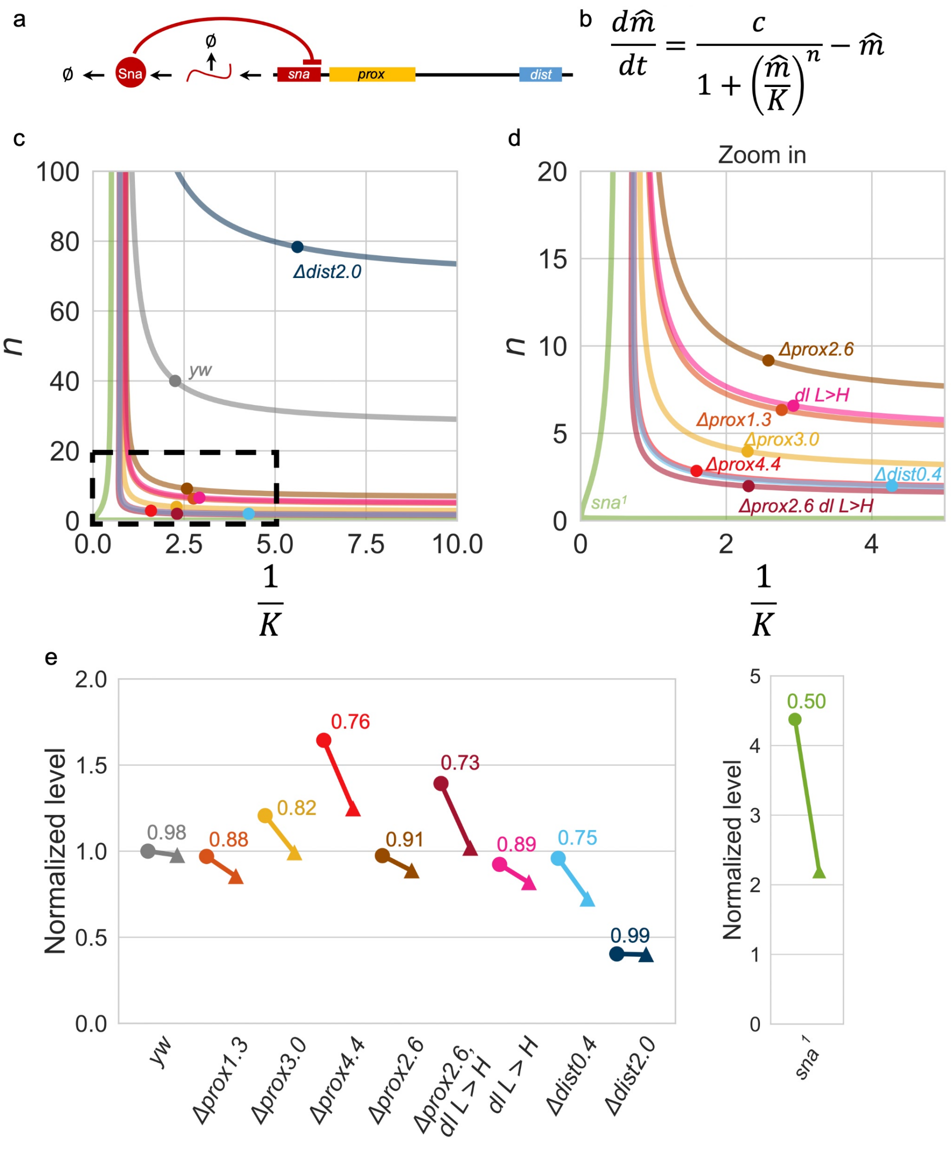

Figure S5: Many solutions to the simple negative autoregulatory feedback model are possible when only relative levels are known. (a) A model of negative autoregulation, where the protein product represses transcription. **(b)** The nondimensionalized ODE used to model simple autoregulatory feedback. $\hat{m}$ is the nondimensionalized *sna* concentration, *c* is copy number, *K* is the apparent binding affinity of Sna, and *n* is the Hill coefficient. **(c)** The values of the parameters 1/*K* and the

Hill coefficient, *n*, that result in specific ratios of one to two copies (lines) that were calculated from Figure 3 for each of the genotypes. From a starting value of *yw* a set of parameters was calculated using the ratio of mutant expression over control expression for each condition (filled circles). The *Δprox4.4* (red) largely overlaps the *Δdist0.4* (cyan). **(d)** Magnified view of the dashed rectangle on the graph in b. **(e)** The *sna* levels that result from the simple negative autoregulation model (eq. 1-16) for the parameters shown with circles in b and c corresponding to their respective conditions. The scale for the sna^1 plot is much larger than the rest of the conditions. In d, the circles represent the *sna* levels for two copies and the triangles represent the *sna* levels for one copy. The levels at one and two copies are connected by a solid line. The numbers above the pairs of data are the ratio of the one copy over two copies. The values in d are normalized by dividing by the steady state value of the control, *yw*, in the two copies condition.

Figure S6

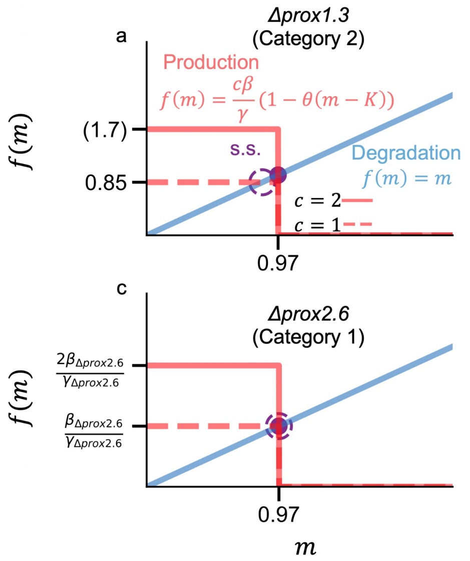

Figure S6: Graphical steady state analysis for genotypes not shown in Figure 4. The same graphical steady state analysis as in Figure 4. The intersection of the step function (red) and straight line (blue) is the steady state solution. In the equations, *m* is the concentration of *sna* mRNA, *θ* represents the Heaviside function or step function, *β* is the production rate of *sna* mRNA, *c* is the copy number, γ is the degradation rate of *sna* mRNA, *n* is the Hill coefficient, and *K* is the threshold of repression. The one copy condition is shown by a dotted line and the two copies condition is shown by a solid line. The graphical steady state analysis for the **(a)** *Δprox1.3* mutation, **(b)** *Δprox3.0* mutation, **(c)** *Δprox2.6* mutation, and **(d)** *dl L>H* mutation. The *Δprox3.0* has a small increase in *K*, and the *Δprox1.3*, *Δprox2.6*, and *dl L>H* are all similar to *yw* (Figure 4g). As in Figure 4, the values of *cβ*/γ and *K* are displayed on the axes. The displayed numbers are found from the data listed in Table S1 in File S2. When the data is not known a variable name is displayed. If the data is inferred, such as the value of 2*β*/γ, the value is displayed in parentheses.

Figure S7

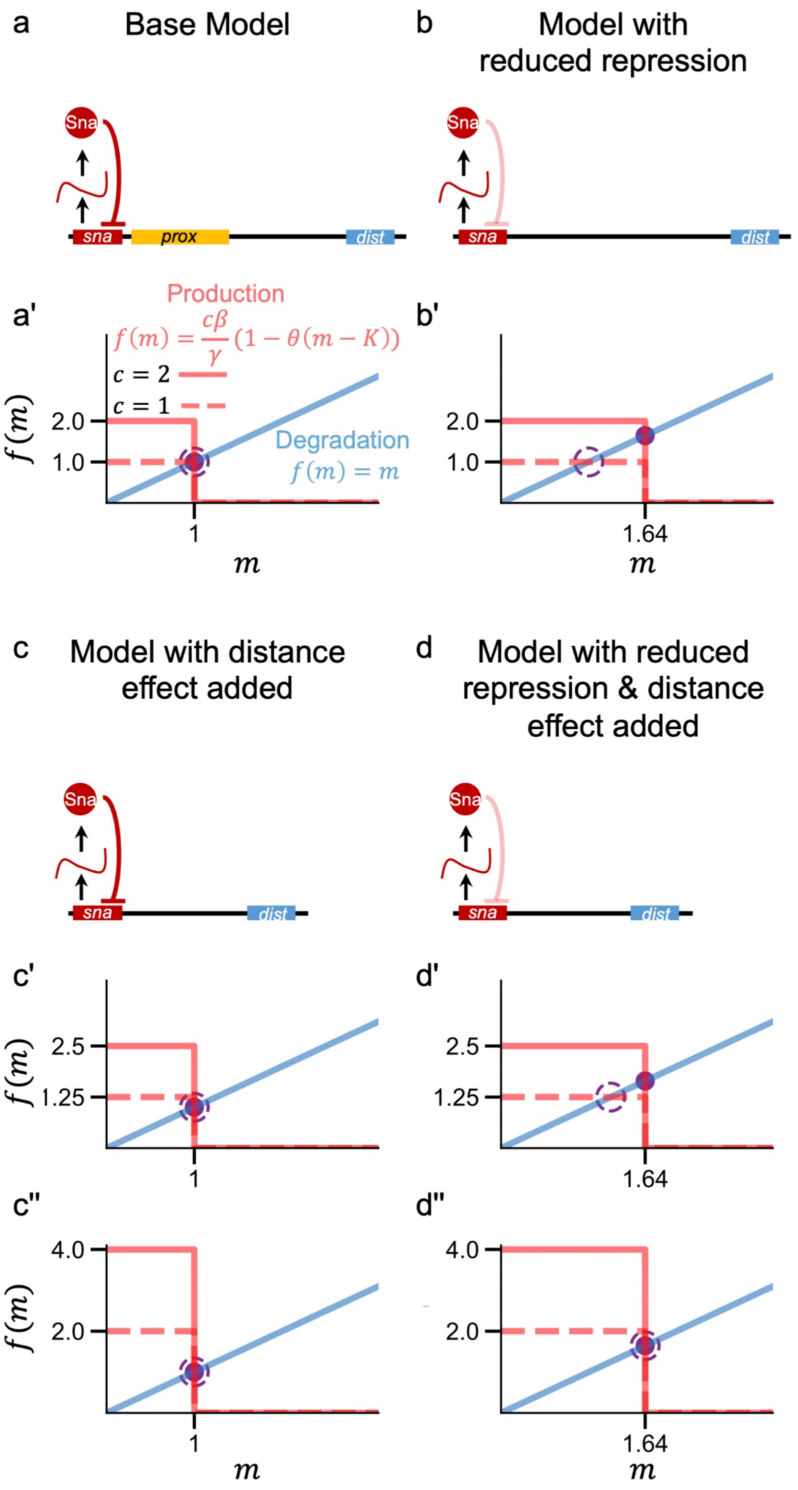

a
Base Model
b
Model with reduced repression
Sna
sna
prox
dist
a'
Production
$f(m) = \frac{c\beta}{\gamma}(1 - \theta(m - K))$
$c = 2$
$c = 1$
2.0
1.0
$f(m)$
Degradation
$f(m) = m$
1
m
b'
2.0
1.0
1.64
m
c
Model with distance effect added
d
Model with reduced repression & distance effect added
c'
2.5
1.25
$f(m)$
1
d'
2.5
1.25
1.64
c''
4.0
2.0
$f(m)$
1
m
d''
4.0
2.0
1.64
m

Figure S7: Moving the distal element closer to the promoter alone is unable to account for changes in *Δprox4.4*. **(a)** A theoretical model of *sna* activation, similar to the control condition (*yw*). **(a')** The graphical steady state solution reproduced from Figure 4g where the steady state value is the intersection between the step function (red) and the straight line (blue). The solid red line is the two copies condition and the dashed red line is the one copy condition. In the equations, *m* is the concentration of *sna* mRNA, *θ* represents the Heaviside function or step function, *β* is the production rate of *sna* mRNA, *c* is the copy number, γ is the degradation rate of *sna* mRNA, and *K* is the threshold of repression. *K* (x-intercept of red step functions) was assigned a value of one, the same value as *yw*. The *β*/γ (y-intercept of dotted red step function) was also assigned a value of one, which is the minimum value *β*/γ can be. **(b)** A model where deletion of the proximal element results in a reduction in repression but does not affect the production rate. **(b')** The graphical steady state solution for b when *K* is increased, but *β*/γ is the same as a'. **(c)** An alternative model where deletion of the proximal element increases the production rate by moving the distal element closer to the promoter, but repression is unaffected. **(c')** The graphical steady state solution for c when *K* is the same as a', but *β*/γ is increased to represent increased production due to the distal element moving closer to the promoter. **(c'')** A similar graphical steady state solution as in c', except *β*/γ is further increased. **(d)** An alternative model where deletion of the proximal element leads to both reduced repression as well as an increase in production due to the distal element being moved closer to the promoter, combining the effects of b and c. **(d')** The graphical steady state solution for d when *K* is increased using the value from b' and *β*/γ is increased using the value from c'. **(d'')** A similar graphical steady state solution as in d', except *β*/γ is further

increased, using the value from c''. The models in which repression is reduced, b and d, more closely resemble the data than the distance only effect of moving the distal element closer to the promoter, c. Thus, a change in the distance of the distal element alone is not able to explain the observed data. Further experimentation is needed to definitively determine if reducing repression alone is sufficient to explain the observed differences or if both reducing repression and a distance effect is occurring.

Figure S8

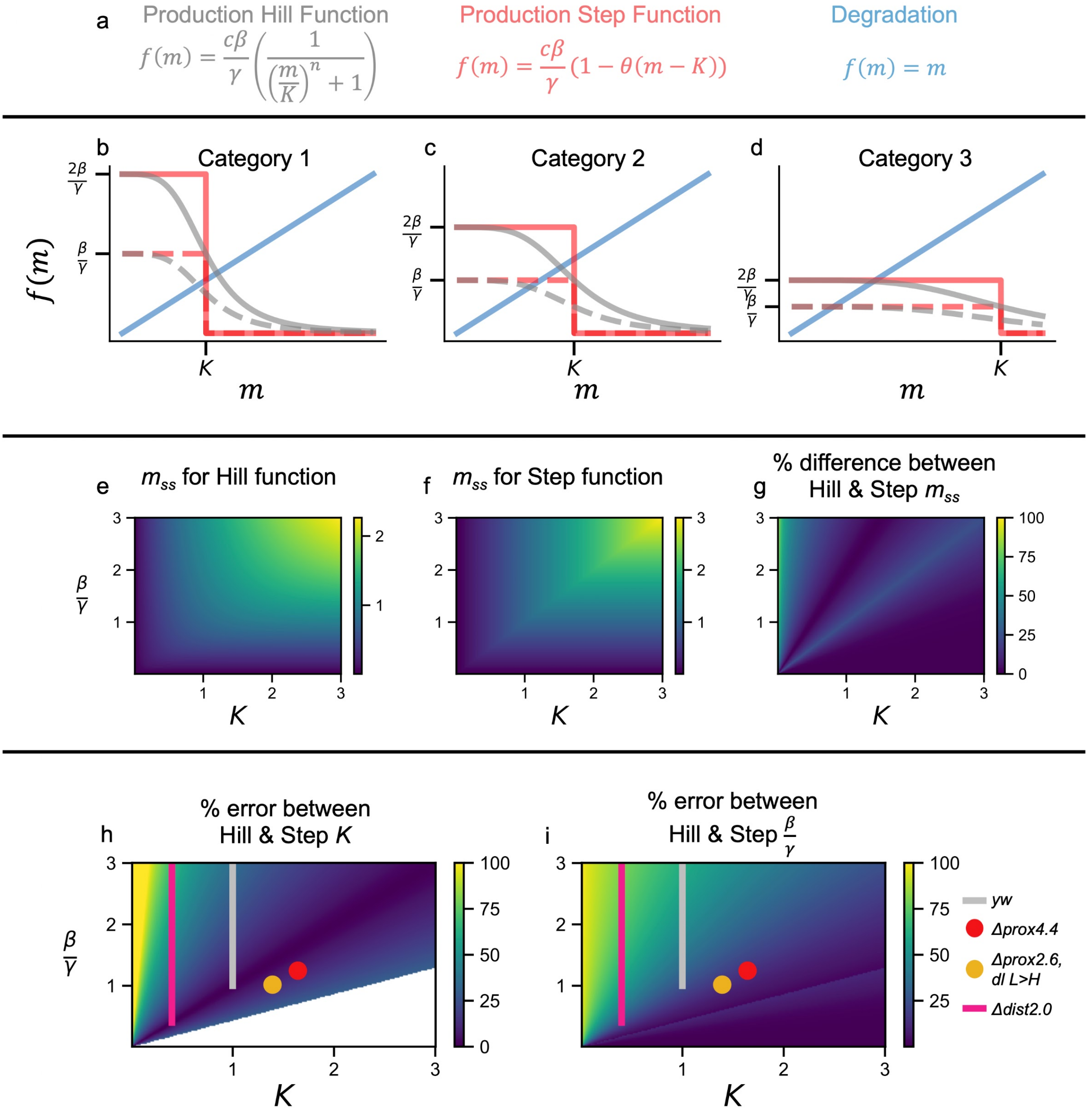

Figure S8: Comparison of Hill function with cooperativity of four to step function.

(a) The equations for a Hill function (gray), step function (red), or straight line (blue), where *m* is the concentration of *sna* mRNA, *θ* represents the Heaviside function or step function, *β* is the production rate of *sna* mRNA, *c* is the copy number, γ is the degradation rate of

sna mRNA, *n* is the Hill coefficient, and *K* is the threshold of repression or the concentration at which the production rate is at half its maximum. **(b,c,d)** Plots of the Hill function with *n* equal to four (gray), a step function (red), and a straight line (blue) where the intersection of the blue line with the red or gray line is the steady state value. The dotted red and gray lines represent one copy and the solid red and gray lines represent two copies. The *K* and β/γ vary between b, c, and d to give the three categories shown in Figure 4d-f. **(e)** The steady state values (m_{ss}) for the Hill function when *n* is four and *K* and β/γ is varied. **(f)** Similar to e, except for the step function. **(g)** The percentage difference between the steady state values (m_{ss}) of the Hill function and step function as *K* and β/γ is varied. The percentage difference is low except when β/γ is high and *K* is low. This gives an estimate of how different the steady state values (m_{ss}) are when *K* and β/γ are known. **(h)** To evaluate how the procedure performed in Figure 4 would affect the estimate of *K* and β/γ, the steady state values were calculated for the Hill function with a *n* of 4 across many *K* and β/γ. The same procedure as in Figure 4 was applied to the steady state values calculated from the Hill function and *K* and β/γ were estimated from a step function. Specifically, if the one to two copies ratio was greater than 0.9 it was assigned to Category 1, if the one to two copies ratio was between 0.6 and 0.9 it was assigned to Category 2, and if the one to two copies ratio was less than 0.6 it was assigned to Category 3. *K* and β/γ were defined as previously described. The percentage error between the known *K* from the Hill function, and the estimated *K* from the step function were calculated and plotted when the known *K* and β/γ were varied. The white space represents areas where this procedure cannot estimate *K* (Category 3) or β/γ (Category 1). **(i)** Similar to h, except the percentage error for β/γ was plotted instead. In

both h and i, the values of K and β/γ are plotted for *yw* (gray line, β/γ is not known), *Δprox4.4* (red circle), *Δprox2.6_dl L>H* (yellow circle), and the *Δdist2.0* (magenta line, β/γ is not known). While the assumption that n goes to infinity is too stringent to give the exact values of K and β/γ, as can be seen from the plots in h and i, the error on these values is relatively low, especially for the *Δprox4.4* and *Δprox2.6_dl L>H*.
